# A unified semantics for distributive and non-distributive universal quantifiers across languages

**DOI:** 10.1007/s11049-025-09673-5

**Published:** 2025-07-09

**Authors:** Nina Haslinger, Alain Noindonmon Hien, Emil Eva Rosina, Viola Schmitt, Valerie Wurm

**Affiliations:** 1 Department of Linguistics and Philosophy, Massachusetts Institute of Technology, 77 Massachusetts Avenue, Cambridge, MA 02139 USA; 2 Leibniz-Zentrum Allgemeine Sprachwissenschaft, Pariser Straße 1, 10719 Berlin, Germany; 3 University Lédéa Bernard Ouedraogo, POBox: 01 BP 346, Ouahigouya, Yatenga Burkina Faso; 4 Institute of Philosophy II, Ruhr University Bochum, Universitätsstraße 150, 44801 Bochum, Germany; 5 Department of Linguistics, University of Vienna, Sensengasse 3a, 1090 Vienna, Austria

**Keywords:** Universal quantification, Distributivity, Non-distributivity, Number, Morphosemantics

## Abstract

Universal quantifiers differ in whether they are restricted to distributive interpretations, like English *every*, or permit non-distributive interpretations, like English *all*. This interpretational difference is traditionally captured by positing two unrelated lexical entries for distributive and non-distributive quantification. But this lexical approach does not explain why distributivity correlates with number: cross-linguistically, distributive universal quantifiers typically take singular complements, while non-distributive quantifiers consistently take plural complements. We derive this correlation by proposing a single lexical meaning for the universal quantifier, which derives a non-distributive interpretation if the restrictor predicate is closed under sum, but a distributive interpretation if it is quantized. Support comes from languages in which the same lexical item expresses distributive or non-distributive quantification depending on the number of the complement. For languages like English that have different expressions for non-distributive and distributive quantification, we propose that the distributive forms contain an additional morphosyntactic element that is semantically restricted to combine with a predicate of atomic individuals. This is motivated by the fact that in several languages, the distributive form is structurally more complex than the non-distributive form and sometimes even contains it transparently. We further show that in such languages, there are empirical advantages to taking the choice between distributive and non-distributive quantifier forms to be driven by semantic properties of the restrictor predicate, rather than morphosyntactic number.

## Introduction and outline

Cross-linguistically, many languages have two (or more) expressions for DP-internal universal quantifiers (UQs), which seem to differ semantically in a uniform way (Gil 1995; Keenan and Paperno 2012, 2017 a.o.). In English, for example, the UQ *every* is restricted to a distributive interpretation, (1a): When *every boy* combines with a predicate like *ate 20 sausages (in total)*, the sentence can only express that this property holds of each boy separately; furthermore, such DPs are incompatible with collective predicates like *met in the yard*. In contrast, the English UQ *all* also permits a non-distributive interpretation, (1b): Combining *all the boys* with *ate 20 sausages (in total)* allows for a cumulative reading (where the number of sausages eaten by the boys add up to 20) and is compatible with collective predicates.

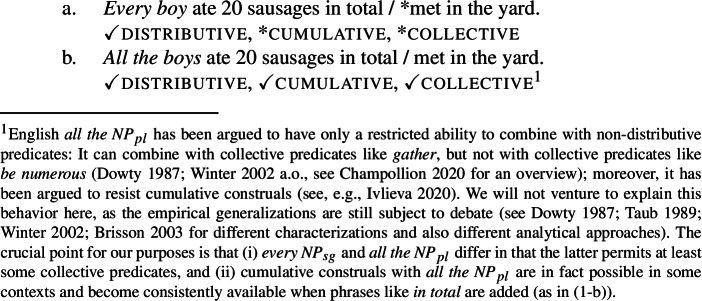

In languages with overt number marking on the NP/DP complements of quantifiers (‘*number languages*’), this difference in interpretation correlates with the morphosyntactic number of the complement: while UQs limited to distributive interpretations tend to occur with singular complements, e.g., *boy* in (1a), UQs permitting non-distributive interpretations usually occur with plural complements, e.g., *boys* in (1b).^2^

Based on this observation, Gil (1995) proposes the generalization that if two UQs in the same language differ both in terms of the readings they permit and in terms of the number of their complements, then distributivity correlates with singular complements and non-distributivity with plural complements. Gil ties this generalization to a ‘markedness’ asymmetry, assuming that singular UQs like English *every* are ‘more marked’ than plural UQs like English *all* in two respects. First, the meaning of *all* is less specific than that of *every*, since *all* is lexically underspecified between distributive and non-distributive quantification, whereas English *every* is purely distributive. Second, Gil assumes that *every* consists of two semantic components, the universal force—the ‘*all*-part’—and an additional distributive component. Since Gil does not provide a compositional analysis, the question arises whether the assumed asymmetry in semantic complexity is reflected in the syntax.

In this paper, we discuss an extended set of cross-linguistic data on number and UQs, partly novel but mostly taken from the literature, especially the diverse sample of languages in Keenan and Paperno (2012), Paperno and Keenan (2017). Based on this dataset, we propose an account for UQs that extends Gil’s proposal, but deviates from it in several respects. In particular, it captures the following four observations:

*Observation 1*: Focusing on languages that exhibit both singular and plural under quantifiers (as languages that neutralize number in these contexts will not be informative^3^), the cross-linguistic pattern seems more categorical than Gil’s 1995 generalization suggests: Within number languages, UQs with singular count NP complements are always *restricted* to a distributive interpretation, while UQs with plural complements are rarely restricted to a distributive interpretation. (We argue in Sect. 8 that some *prima facie* counterexamples are in fact compatible with a strict correlation between singular complements and distributivity.)

*Observation 2*: Several unrelated languages have strategies where non-distributive and distributive quantification are expressed by the same lexical item: the quantifier receives a distributive interpretation if its complement is singular, and a non-distributive one if its complement is plural. This motivates a single primitive quantifier meaning, instead of two distinct meanings as proposed by Gil 1995.

*Observation 3*: Cross-linguistically, UQs that are limited to a distributive interpretation tend to be morphosyntactically more complex than those that permit non-distributive construals. This supports the idea of a syntactic asymmetry between the two forms.

*Observation 4*: The distribution of such obligatorily distributive UQ forms is narrower than the distribution of singular morphology: In languages where a UQ form can be used to quantify over mass parts or over parts of an atomic individual (contributing the meaning of English *whole*), it is consistently the non-distributive form. This suggests that the choice between UQ forms is conditioned by semantic properties of the complement, not directly by morphosyntactic number.

Taking these observations at face value, our proposal is as follows: There is a single semantic operator for universal quantification, $\mathcal{Q}_{\forall }$, cross-linguistically. $\mathcal{Q}_{\forall }$ applies its nuclear-scope predicate to every maximal element of the restrictor denotation. If the restrictor predicate is a singular count NP, this entails applying the predicate to all the atoms in the noun extension; if it is plural, the predicate is applied to the maximal plurality in the noun extension. Thus, the difference in interpretation is a result of the combination of $\mathcal{Q}_{\forall }$ with the respective complement meaning. This approach derives the correlation between distributivity and number observed by Gil (1995), but goes beyond his description in providing a compositionally interpreted syntax for UQs that rules out the unattested number-interpretation combinations.

On this view, strategies where one and the same lexical item is used to express distributive and non-distributive quantification are expected and in fact the default case. To account for languages with distinct distributive and non-distributive UQ forms, we assume that the distributive form has a complex underlying syntactic structure. This structure consists of $\mathcal{Q}_{\forall }$ and an additional syntactic element, which we dub one due to its semantic similarity with the numeral *one*, and which is incompatible with plural or mass complements. Support for this comes from languages where the distributive forms transparently contain morphemes formally identical to the numeral *one*. Non-distributive forms like English *all*, in contrast, are realizations of $\mathcal{Q}_{\forall }$ in the absence of one.

We therefore follow Gil’s intuition that distributive UQs are decomposable, but interpret it more literally: On our account, the primitive quantificational element $\mathcal{Q}_{\forall }$ is exactly the same in distributive and non-distributive UQs, but distributive forms like *every* are portmanteau realizations of $\mathcal{Q}_{\forall }$ and the additional syntactic head one. Unlike Gil (1995), we thus capture the cross-linguistic morphosyntactic complexity asymmetries between distributive and non-distributive forms without having to assume that one of them has a ‘less marked’ meaning than the other. At first glance, the non-distributive forms seem to be semantically underspecified in the sense that they are compatible with distributive interpretations as well. However, we will argue that in such cases distributivity is not introduced by the quantifier, but by a VP-level distributivity operator (Link 1987 a.o.) that must be realized overtly in some languages.

Overall, our approach to the syntax and semantics of UQs thus builds on two methodological choices (cf. the typology of accounts of ‘logical words’ proposed by Szabolcsi 2010, 202): First, we use semantic and syntactic decomposition below the word level, and in doing so take the presence of extra overt material to constitute evidence for a more complex syntactic structure. Second, we aim to develop a syntactic and semantic account that is uniform across languages where plausible, even if this means positing complex syntactic structures whose primitive elements are not transparently realized in every language (for example, we decompose English *every* syntactically although it is synchronically intransparent).

The paper is structured as follows: Sect. 2 presents the empirical situation motivating our distributivity-number generalization (DNG): the interpretation of a universal quantifier seems to be driven by the number of its restrictor complement. Section 3 shows that standard assumptions about the semantics of morphological number on nouns, combined with a standard semantics for *every*-type and *all*-type quantifiers, fail to derive the DNG. Section 4 provides some background on plural semantics and presents our proposal: there is a single lexical meaning for universal quantifiers cross-linguistically that derives the correlation between complement number and distributivity. Section 5 extends the proposal to strategies using distinct distributive and non-distributive UQ forms. In Sect. 6, we refine the proposal in order to capture the interaction of UQs with numeral-modified NPs and definite plural DPs, as well as some differences between distributive UQs, such as English *every* vs *each*. In Sect. 7, we discuss instances of UQ with mass complements and singular DP complements, which suggest that the choice between UQ forms is conditioned by semantic properties rather than morphosyntactic number, and compare our account to existing work on variants of the DNG by Winter (2001) and Fassi Fehri (2020). Section 8 discusses potential counterexamples to the DNG and whether they can be analyzed in a way that is compatible with the generalization. Section 9 concludes the paper and points to some open issues.

## Empirical situation: A novel generalization

We first provide a partial overview of the empirical situation regarding attested combinations of quantifier interpretation and complement number. We then introduce a strengthened variant of an empirical generalization suggested by Gil (1995), based on data from the literature and new data from Mabia languages. To simplify the exposition, our claims about non-plural complements are restricted to singular count NPs for now; we will discuss mass and singular DP complements in Sect. 7.

### Two forms for universals

We start with the observation that the pattern presented in (1) is not idiosyncratic to English: in several languages with two formally different DP-internal UQs (henceforth ‘2-form languages’), the latter differ both syntactically, i.e., with respect to the number of the complements they permit, and semantically, i.e., concerning the availability of distributive and non-distributive readings.

We will focus on UQs in subject position, as schematized in (2) (XP stands for the constituent introducing the UQ’s restriction).^4^ A UQ will be said to permit a distributive interpretation if the sentence is true in scenarios where the property expressed by the VP holds of each atomic XP-individual separately. And a UQ will be said to permit a non-distributive interpretation if (i) the UQ can combine with collective predicates, or (ii) cumulative construals are possible, which, intuitively speaking, means that we ‘add up’ properties of the XP-individuals, so that the property expressed by the VP holds of the plurality of all XP-individuals as a whole and not of each atomic part separately.

(2)

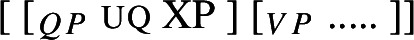

Example (3) shows that the two German UQs *jed-* and *all-* differ regarding their compatibility with collective predicates: while *all-* can combine with such predicates, (3b), *jed-* cannot, (3a).

(3)
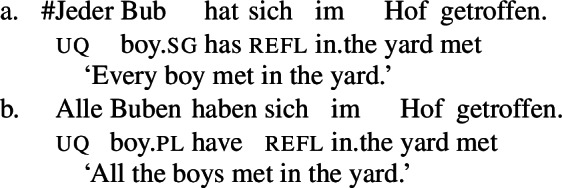

Moreover, *all-* allows for a cumulative construal, but *jed-* does not: While (4b) can be judged true in the cumulative scenario (5-a), (4a) is false in this scenario. Finally, both *all-* and *jed-* permit a distributive construal—both (4a) and (4b) are true in the distributive scenario (5-b).^5^

(4)

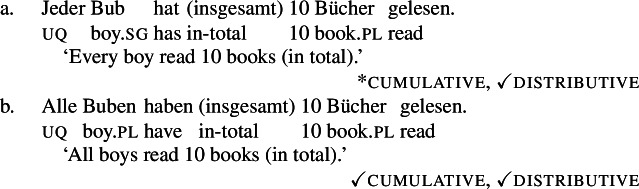

(5)
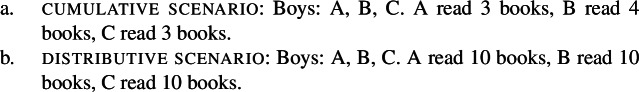

From now on, we refer to UQs such as German *all-*, which are compatible with non-distributive construals as [−dist], and to those that are obligatorily distributive, such as German *jed-* as [+dist]. (The values [+dist] and [−dist] are not symmetric: [−dist] indicates compatibility with non-distributive readings (collective predicates and/or cumulativity), but does not indicate whether the element in question also permits a distributive reading.)

Across number languages, these semantic differences correlate with a syntactic difference: the elements restricted to a distributive construal ([+dist] elements), e.g., German *jed-* in (6), tend to take singular NP complements, while the elements permitting a non-distributive reading ([−dist] elements), e.g., German *all-* in (7), tend to take plural NP or plural DP complements.^6^

(6)

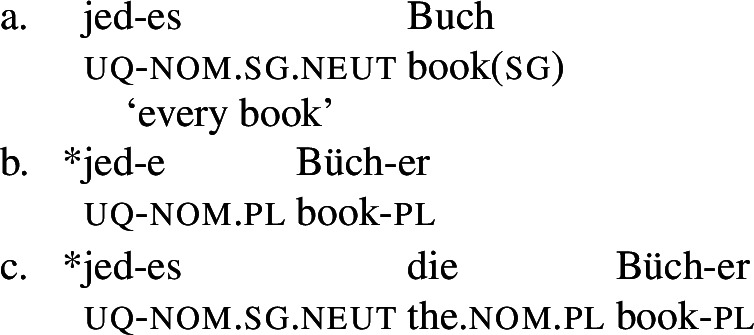

(7)
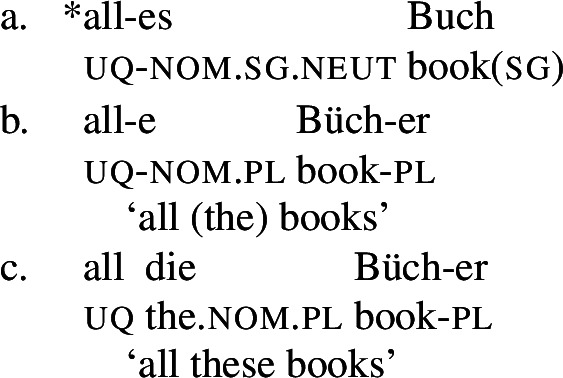

As noted above, Gil (1995) already observes this correlation between the interpretation of a quantifier and the number of its potential complements. He suggests the following implicational universal:

(8) If distributive and non-distributive UQs of a certain language differ with respect to the number of the complement they permit, then the distributive UQ requires a singular complement and the non-distributive UQ a plural complement.

(8) does not exclude languages in which distributive and non-distributive UQs both take plural complements, or both take singular complements. It therefore does not rule out the combination of a singular complement with non-distributivity, or of a plural complement with distributivity. But do we actually find such combinations, or can the generalization be strengthened? To answer this question, we considered a sample involving both data from the literature (Keenan and Paperno 2012; Paperno and Keenan 2017; Landman 2016; Zimmermann 2008) and novel data we elicited.

Our first crucial observation is that the form-meaning correspondence observed for German and English extends to several other Indo-European and non-Indo-European languages. Table 1 gives some examples. Note that in Hindi and Russian, the non-distributive UQ forms are syntactically compatible with singular complements, but the resulting semantics is that of English *whole*, rather than a quantifier over individuals like English *all* or *every*. We put these uses aside for now and return to them in Sect. 7.2. Similarly, Landman (2016) reports that in Logoori the distributive UQ can take plural complements, but only with a special reading involving distribution over groups, which we return to in Sect. 6.1.

**Table 1:** Languages with [+dist]-singular and [−dist]-plural universal quantifier

language	source	form	distributivity	sg complement?	pl complement?
English (Indo-European, Germanic)		*every*	[+dist]	✓	✕
German (IE, Germanic)		*jed-*	[+dist]	✓	✕
Hindi (IE, Indic)	Mahajan (2017)	*praty-ek*	[+dist]	✓	✕
Russian (IE, Slavic)	Paperno (2012)	*každyj*	[+dist]	✓	✕
Imbabura Quichua (Quechuan)	Barchas-Lichtenstein et al. (2017)	*kada*	[+dist]	✓	✕
Turkish (Turkic)	Özyıldız (2017)	*her*	[+dist]	✓	✕
Basque (isolate)	Etxeberria (2012)	*bakoitz*	[+dist]	✓	✕
Telugu (Dravidian)	Ponamgi (2012)	*prăti:*	[+dist]	✓	✕
Hausa (West Chadic)	Zimmermann (2008)	*koowànè*	[+dist]	✓	✕
Logoori (Bantu)	Landman (2016)	*vuri*	[+dist]	✓	only ‘subgroup’ meaning
English		*all*	[−dist]	✕	✓
German		*all-*	[−dist]	✕	✓
Hindi	Mahajan (2017)	*saar-*	[−dist]	only ‘whole’ meaning	✓
Russian	Paperno (2012)	*vse*	[−dist]	only ‘whole’ meaning	✓
Imbabura Quichua	Barchas-Lichtenstein et al. (2017)	*tukuy(-lla)*	[−dist]	✕	✓
Turkish	Özyıldız (2017)	*bütün, hepsi*	[−dist]	✕	✓
Basque	Etxeberria (2012)	*guzti, den, oro*	[−dist]	✕	✓
Telugu	Ponamgi (2012)	*ăndărŭ, ăn:ĭ, ănta:*	[−dist]	✕	✓
Hausa	Zimmermann (2008)	*duk*	[−dist]	✕	✓
Logoori	Landman (2016)	*-oosi*	[−dist]	✕	✓

By ‘sg complement’ we mean singular count NPs; by ‘pl complement’ we mean plural NPs or plural DPs.

More generally, excluding languages that do not mark number on complements of quantifiers at all, only two of the four logically possible number-distributivity combinations are widely attested: [+dist] UQs (i.e., UQs that are obligatorily distributive) with singular complements, and [−dist] UQs (i.e., UQs that can receive a non-distributive interpretation) with plural complements.

[−dist] UQs with a singular complement (and an individual-quantifier meaning as opposed to a ‘whole’ meaning) are not attested in our sample. The sample does contain a few languages in which a [+dist] quantifier seems to take a plural complement. Yet, in Sect. 8, we will argue that most of these cases are susceptible to plausible alternative analyses compatible with the generalization that [+dist] quantifiers with a plural complement are ruled out.

### One form for the universal

Our second crucial observation is that some languages—like Dagara, Moore, and Gourmantchema (Mabia), Wolof (Atlantic; Tamba et al. 2012) or Arabic (Semitic; e.g., Fassi Fehri 2020)—have a single lexical item that can express both distributive and non-distributive quantification depending solely on the number of the complement it combines with (“1-form languages”):^7^ in these languages, there is a single UQ form that is used both in constructions expressing distributive quantification and in those expressing non-distributive quantification. Crucially, we find the same correlation between number and interpretation as in 2-form languages: if the complement is singular, the result is [+dist] universal quantification; if it is plural, we obtain [−dist] universal quantification. Thus in 1-form languages the interpretation is exclusively determined by the semantic number of the restrictor complement.

Take Dagara: the UQ *’hà* yields [+dist] universal quantification when combined with a singular NP complement (*’hà*+NP_sg_): (9a) is true in a distributive scenario, (10-b), but false in a cumulative scenario, (10-a). In contrast, combining *’hà* with a plural DP complement (*’hà*+DP_pl_) yields [−dist] universal quantification: (9b) can be true in a cumulative scenario, (10-a), but is false in a distributive scenario, (10-b):

(9)

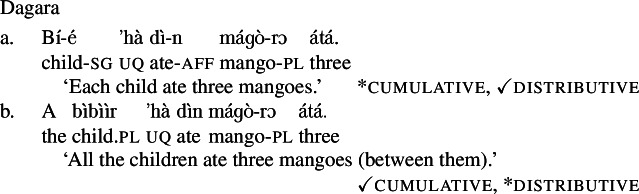

(10)

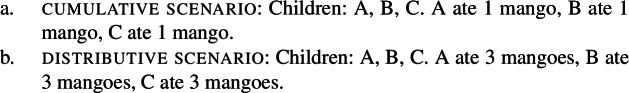

While it is possible to construct sentences with the *’hà*+DP_pl_-strategy that have a distributive interpretation, this requires distributivity marking on the predicate, i.e., an extra pl-marker on the lower numeral DP. This is exemplified in (11), which is true in a distributive scenario like (10-b), but false in a cumulative scenario like (10-a). Without distributivity marking on the predicate, e.g., in (9b), a distributive construal is unavailable, so the *’hà*+DP_pl_ strategy cannot be said to be underspecified between distributive and non-distributive interpretations.

(11)

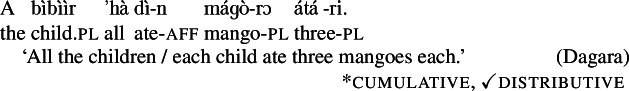

The same pattern is observable in other Mabia languages: in Moore and Gourmantchema, the single lexical items *fãa* and *kuli*, respectively, yield [+dist] universal quantification with singular NP complements, and [−dist] universal quantification with plural NP or DP complements ((12b) and (13-b) are true in the cumulative scenario (10-a), but not in the distributive scenario (10-b)). To express a distributive interpretation with a plural complement, extra distributivity marking within the lower numeral DP is necessary, (12c) and (13-c).

(12)

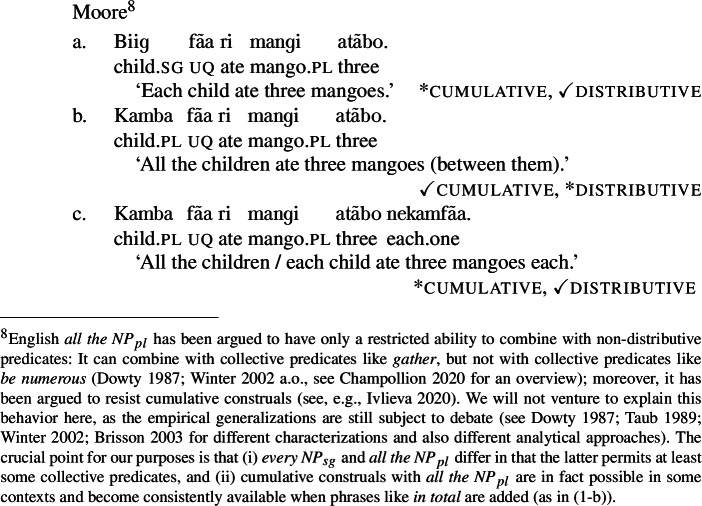

(13)
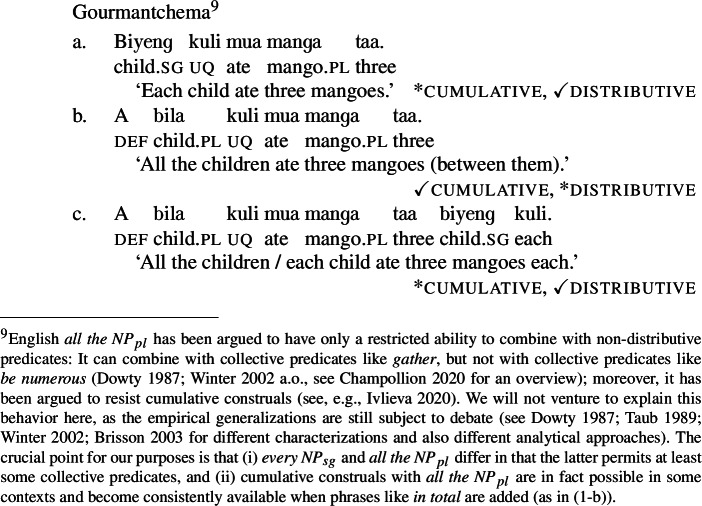

According to Tamba et al. (2012), the Wolof universal *-epp* exhibits the same pattern. It is [+dist] when combining with a singular noun (14a), but [−dist] when combining with a plural noun or plural DP (14b),^10^ as illustrated with collective predication (15).

(14)

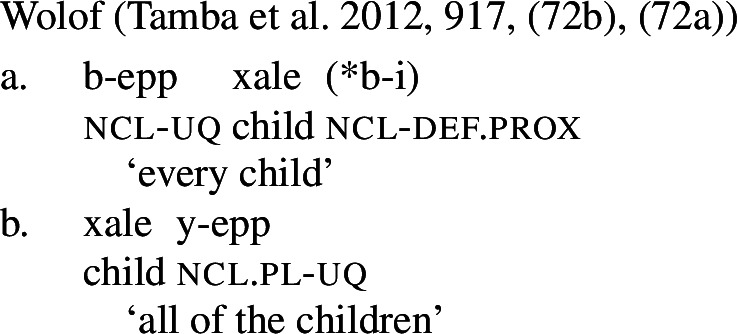

(15)
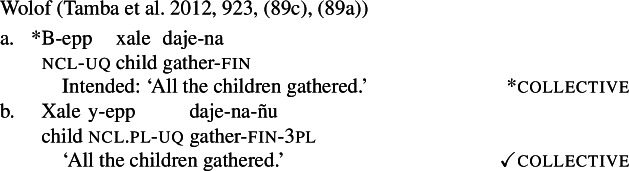

The pattern is also found in Arabic, as discussed by Fassi Fehri (2020) and illustrated in (16) for Syrian Arabic *kul*: *kul*+NP_sg_ is a [+dist] universal, i.e., (16a) can be true in a scenario such as (10-b), but not in a scenario such as (10-a).^11^*kul*+DP_pl_ on the other hand, is a [−dist] universal, i.e., (16b) can be true in (10-a), and also—although dispreferred—in (10-b).

(16)
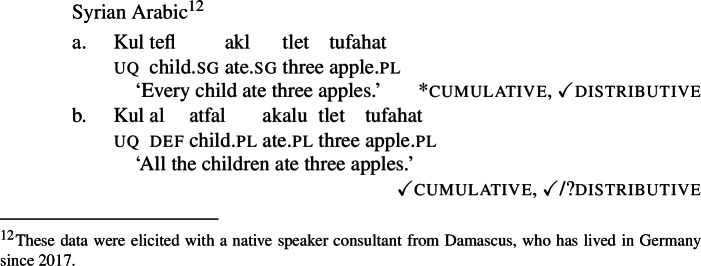

Table 2 sums up the pattern in 1-form languages. The crucial observation is that the number of the complement predicts the availability of non-distributive interpretations (second column). The availability of distributive interpretations is not fully predicted by number (third column), an issue we return to below.

**Table 2:** One element: [+dist] sg and [−dist] pl UQ

language	strategy	non-distributive?	distributive?
Dagara	*’hà*+NP_sg_	✕	✓
Moore	*fãa*+NP_sg_	✕	✓
Gourmantchema	*kuli*+NP_sg_	✕	✓
Wolof (Tamba et al. 2012)	*-epp*+NP_sg_	✕	✓
Syrian Arabic	*kul*+NP_sg_	✕	✓
Dagara	*’hà*+DP_pl_	✓	✕
Moore	*fãa*+NP_pl_	✓	✕
Gourmantchema	*kuli*+DP_pl_	✓	✕
Wolof (Tamba et al. 2012)	*-epp*+NP_pl_/DP_pl_	✓	unknown
Syrian Arabic	*kul*+DP_pl_	✓	✓/?

Besides number, two other formal properties seem to correlate with the availability of non-distributive interpretations. First, in three of the five languages in Table 2, the non-distributive structures obligatorily involve definite DP complements, while the distributive structures consistently involve NP complements (see also Fassi Fehri 2020). Our analysis will take the choice between distributive and non-distributive interpretations to be driven by semantic properties related to number, rather than definiteness. However, we think the correlation with definiteness is not accidental and will return to it in Sect. 6.4.

Second, in Wolof, the quantifier precedes its complement in the [+dist] construction, but follows it in the [−dist] constructions. While we do not think that linear order is directly implicated in distinguishing between [+dist] and [−dist] UQ (in Arabic, UQs precede their complements regardless of distributivity, whereas in the Mabia languages discussed above they always follow their complements), the existence of such word order differences suggests that distributive and non-distributive UQ systematically involve complements of distinct categories. We return to this in Sect. 6.4.

### The distributivity-number generalization

Given this overall cross-linguistic pattern, we propose the following generalization for number languages (see Winter 2001 for a similar claim):

(17)
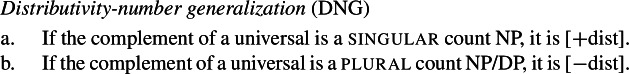

The DNG excludes certain types of UQ strategies in both 1-form and 2-form languages: i) UQs expressing [−dist] universal quantification when they combine with a singular count NP, and ii) UQs expressing [+dist] universal quantification when they combine with a plural complement. (As stated above, we exclude UQs with mass and singular DP complements for the time being.) Note also that we are only considering constructions involving NP and DP complements. Thus, our generalization does not expand to partitive complements, i.e., it is not testable in languages that lack overt partitive marking, as in such languages it would be hard to distinguish a partitive from a “direct” NP or DP complement. (See Sect. 5.6 for more discussion on partitives.)

Generalizations similar to (17) have been proposed in earlier work, especially Gil (1995) and Winter (2001). Winter (2001) notes that across different quantificational constructions found in English, [+dist] interpretations are systematically associated with singular complements, and states that the same correlation holds in “many other languages.” While Winter’s claim is stronger than ours in that it is not restricted to UQ, the question of whether this pattern is uniform across number languages is left open. Similarly, Gil’s 1995 implicational universal does not explicitly exclude [−dist] forms with singular count complements and [+dist] forms with plural complements.

Evidence that the absence of these distributivity/number combinations is systematic comes from the survey in Keenan and Paperno (2012), Paperno and Keenan (2017): Excluding languages in which complements of quantifiers do not exhibit evidence of morphosyntactic number, their diverse cross-linguistic sample contains no counterexamples to (17a). In Sect. 8, we will discuss counterexamples to (17b) and argue that some of them can be given an analysis compatible with the DNG, although we must leave at least one counterexample as an open problem.

Supported by languages like Dagara, Moore or Gourmantchema, where a distributive interpretation with a [−dist] UQ requires extra marking on the predicate ((11), (12-c), (13-c)), we moreover suggest that distributive interpretations of [−dist] UQs result from the presence of distributivity operators in the VP (see, e.g., Link 1987). This additional material can be covert in languages like English and German (see Flor et al. 2017). Thus, the source of distributive interpretations of [−dist] UQs is independent of the semantic contribution of the UQ: [−dist] UQs as such are specified for a non-distributive meaning in the sense that we will get a non-distributive interpretation, unless the VP-predicate contains a distributivity operator. This goes against Gil’s 1995 claim that [−dist] quantification is semantically less marked—i.e., underspecified regarding distributivity—compared to [+dist] quantification. Rather, neither combination of UQ and complement number is underspecified regarding its interpretation.

All of this points to a direct correlation between singular NP complements and distributive meanings, and between plural NP/DP complements and non-distributive meanings. If sentences with UQs with plural complements permit distributive interpretations in addition to the non-distributive ones, the reason for this is the predicate rather than any underspecification of the quantifier.

## The semantic puzzle raised by the DNG

Before presenting a unified semantics for UQs that captures this correlation between their interpretation and the number of their complements, we motivate the need for a new account by arguing that the correlation is unexpected in light of standard assumptions: An otherwise plausible semantics in which *every* is inherently distributive, whereas *all* is a maximizer does not explain the DNG, when combined with a standard semantics for number on nouns.

### Background: Plural individuals and parthood

We first provide the background for our discussion by introducing some basic notions of plural semantics.

We assume that the domain $D_{e}$ contains not only what we would pre-theoretically think of as individuals, but also *sums* or *pluralities* of individuals (we use these two terms interchangeably). We use the symbol $\text{ $+$}$ for the operation that maps any nonempty subset of $D_{e}$ to its sum: $\text{ $+$}(\{x : \textbf{student}(x)\})$ is the sum of all the students, and $\text{ $+$}(\{\text{Ann}, \text{Bert}\})$ is the plurality consisting of Ann and Bert. To avoid clutter, we use the notation *a* + *b* for $\text{ $+$}(\{a, b\})$.

**Fig. 1:**
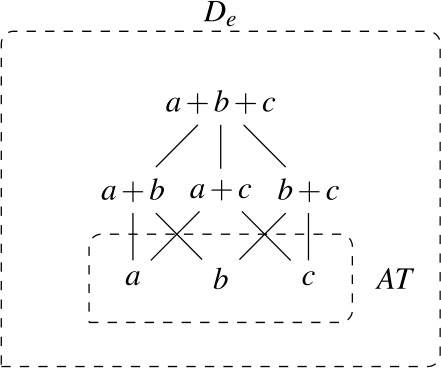
Atomic individuals and pluralities in $D_{e}$

We assume that $D_{e}$ has a proper subset *AT* of *atomic individuals*. For now, we assume that *AT* contains exactly those individuals that are not sums of two or more distinct parts. While we do not technically identify sum individuals with nonempty subsets of *AT* (see, e.g., Link 1983; Schwarzschild 1996 for discussion), we assume a one-to-one correspondence between them, i.e., the structures ($D_{e}$, +) and $(\mathcal{P}({AT}) \backslash \{\emptyset \}, \bigcup )$ are isomorphic. For instance, the sum *a* + *b* corresponds to the set {*a*,*b*}. Due to this correspondence, we can think of $D_{e}$ as having the structure of a complete atomic Boolean algebra with the bottom element removed, as illustrated in Fig. 1. We will have to weaken these ontological assumptions when we discuss mass nouns in Sect. 7.1.

We use the symbol ⊑ for the mereological part-of relation on $D_{e}$, which can be defined as in (18). (For an overview of the use of mereology in semantics, see Champollion and Krifka (2016).)

(18) $\forall x, y \in D_{e}.x \sqsubseteq y$ iff *x* + *y* = *y*.

Finally, our formulas will occasionally make use of a pluralizing *star operator* ^∗^ that attaches to unary predicates (Link 1983). Intuitively, a pluralized predicate ^∗^*P* is true of an individual iff that individual can be expressed as a sum of individuals that satisfy *P*:

(19) Given a predicate *P*: ^∗^*P*(*x*) iff $\exists S \subseteq D_{e}.x = \text{ $+$}(S) \land \forall y[y \in S \rightarrow P(y)]$

### Why the DNG is unexpected under standard assumptions

Usually, singular count nouns are taken to denote sets of atomic individuals (20a), whereas plural nouns denote sets of atomic as well as plural individuals (20b) (see, e.g., the underlying number semantics assumed in Sauerland 2003).^12^

(20)
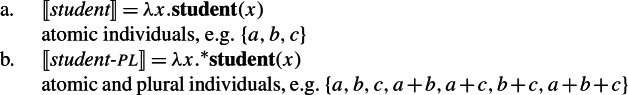

For concreteness, we assume that syntactically, all count nouns come with a projection of a feature # (21), and that plural count nouns contain an additional head pl on top of # (22). We take the extension of the #P to consist of atomic individuals only, but remain neutral on how this comes about (e.g., whether # has semantic content related to the semantics of classifiers). Our general proposal does not hinge on this containment relation between singular and plural; in particular, it is compatible with analyzing languages like Dagara, where this containment is not obvious in terms of a single number projection, as suggested by the surface structure.

(21)

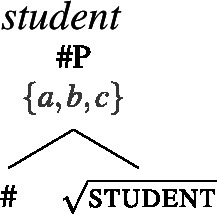

(22)
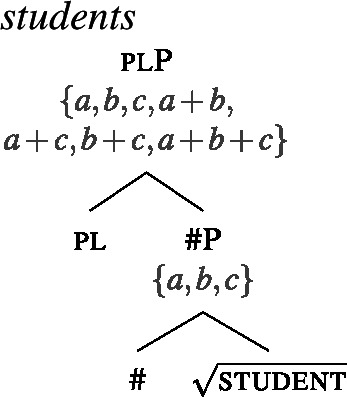

Standardly, *every*-type and *all*-type quantifiers are taken to have distinct lexical entries that are not derivationally related (e.g., Link 1983). This is particularly clear in the recent literature on “non-maximal” readings of plurals (e.g., Brisson 1998; Malamud 2012; Križ 2015), which takes the core semantic function of *all* to be maximization. A simplified variant of this maximality view of *all*, which abstracts away from the implementation details, is given in (23b). While *all* requires its nuclear scope predicate to apply to the sum of all individuals satisfying the restrictor predicate, *every* requires it to apply separately to each such individual (23a).^13^

(23)
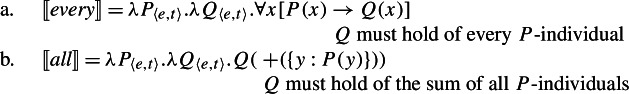

Combining the meaning for *every* with the singular noun denotation thus gives us a distributive quantifier, (24a), while combining the meaning for *all* with the plural noun denotation gives us a non-distributive quantifier, (24b).

(24)
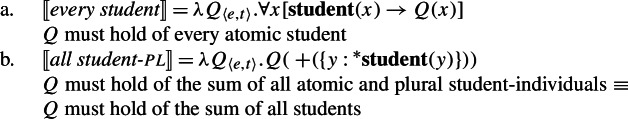

Crucially, under these standard assumptions, there is no semantic reason to expect a complementary distribution based on number, as the two cross-linguistically unattested combinations are also interpretable. First consider the unattested case [−dist]+NP_sg_: (25) shows that there is no reason to expect this pattern to be absent, as combining 〚*all*〛 from (23-b) with a singular noun denotation should yield exactly the same meaning as combining it with a plural noun denotation.

(25) $[\!\![{}\textit{all student-}\textsc{sg}]\!\!]{} = \lambda Q_{\langle{}e, t\rangle{}}.Q$( +({*y*:**student**(*y*)}))
  *Q* must hold of the sum of all students

For the combination [+dist] + NP_pl_, the quantifier meaning in (23-a) together with a standard semantics for plural nouns yields (26). Again, there is no reason why this meaning should be blocked.^14^

(26) 〚*every student-*pl$]\!\!]{} = \lambda Q_{\langle{}e, t\rangle{}}.\forall x[^{*}\textbf{student}(x) \rightarrow Q(x)]$
  *Q* must hold of every atomic student and every plurality of students

One way to account for the DNG, while maintaining the gist of the above assumptions, would be to slightly change the lexical meanings of *all* and *every* so that they semantically select a complement with a certain semantic number. However, this is not only stipulative, but also requires positing an unmotivated lexical ambiguity for the UQ forms in 1-form languages. Accordingly, we propose a novel syntax and semantics for universal quantifiers that derives the correlation between number and interpretation. Our approach treats 1-form languages like Dagara, Moore, Gourmantchema, Wolof, and Arabic as the default case, rather than requiring special assumptions for such languages.

## A uniform semantic primitive for universal quantification

We suggest that the existence of 1-form languages (where the same UQ form receives a [+dist] interpretation if the complement is singular and a [−dist] interpretation if the complement is plural) motivates a single underlying meaning for universal quantification:

(27)
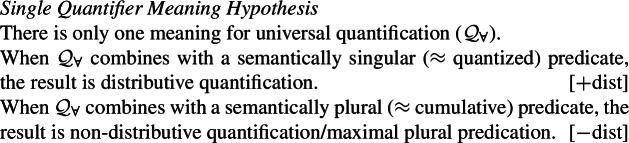

**Fig. 2:**
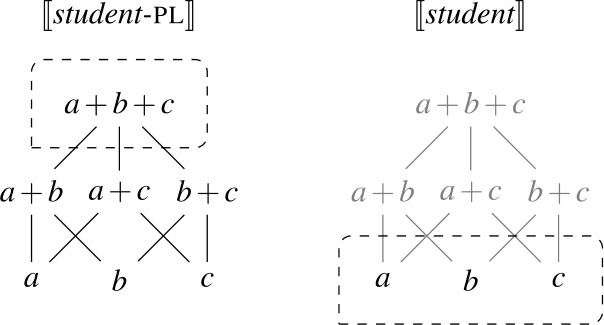
Maximal elements of plural and singular noun extensions

As a first step towards specifying $\mathcal{Q}_{\forall }$, we note that the extensions of singular and plural nouns differ in their algebraic structure. Consider the definition of maximality in (28), according to which an element *x* is maximal in a set *S* only if *S* does not include an element of which *x* is a proper part. Given (28), singular noun denotations consist exclusively of maximal elements, while plural noun denotations have a single maximal element, as illustrated in Fig. 2:

(28) For any set *S*, *x* is maximal in *S* iff *x*∈*S*∧¬∃*y*∈*S*[*x*⊏*y*].

Exploiting this structural difference between singular and plural noun denotations, we assume that the morpheme $Q_{\forall}$—the object language correlate of $\mathcal{Q}_{\forall }$ in (27)—has the denotation in (29): $[\!\![{}\textit{$Q_{\forall }$}]\!\!]{}$ (i.e., $\mathcal{Q}_{\forall }$) applies its scope argument to every maximal element of the noun denotation. While we will revise the proposal below, this semantic intuition will be preserved. (*α* is a variable over types so as to permit UQs to combine with predicates of other types for which a part-whole relation is defined.)

(29)
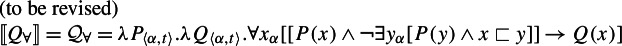

This lexical meaning immediately derives the DNG for count NP complements: When $\mathcal{Q}_{\forall }$ combines with a plural noun denotation as in (30a), its semantic effect is maximization without any distributive inferences—the only maximal element in 〚*student-*pl〛 is the plurality of all students, so the scope property has to hold of this plurality. This derives the attested pattern [−dist]+NP_pl_. When $\mathcal{Q}_{\forall }$ combines with a singular noun denotation as in (30b), the same requirement will yield distributivity—all the atomic individuals in 〚*student*〛 are maximal, so the scope property must hold of each of them separately. This yields the attested pattern [+dist]+NP_sg_. Crucially, the two unattested patterns [−dist]+NP_sg_ and [+dist]+ NP_pl_ are not derivable within this account.

(30)
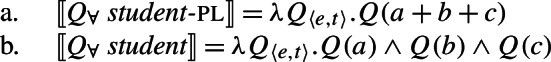

The proposal can also be extended to account for the cross-linguistically common tendency to mark distributive conjunctions using morphemes that occur in UQs ((31); see, e.g., Szabolcsi 2010; Jayaseelan 2011; Mitrović and Sauerland 2014, 2016; Szabolcsi 2015; Haslinger et al. 2019 a.o.). Mitrović and Sauerland (2014) and Haslinger et al. (2019) show (using different frameworks) that the distributive effects of conjunctions involving UQ morphemes can be accounted for if the semantic job of these morphemes is simply to shift each individual conjunct to a quantificational type. This is exactly what $Q_{\forall}$ does if its restrictor predicate is a singleton. Our proposal therefore extends to occurrences of UQ morphemes in conjunctions, although we do not spell out the details here due to space limitations.

(31)
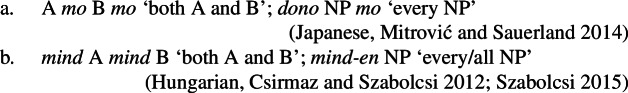

One aspect of the DNG that this proposal does not derive yet is the possibility (which in many languages is a requirement, cf. Matthewson 2001) for [−dist] UQs to take a DP rather than an NP complement. We return to this issue in Sect. 6, once we have given an account of 2-form languages.

## The morphosemantics of distributive quantifier forms

We have developed a uniform account of [+dist] and [−dist] UQs in 1-form languages like Dagara, based on a single quantifier meaning $\mathcal{Q}_{\forall }$. But how can we extend this approach to 2-form languages like German or English?

Intuitively, it seems plausible to view the formal variation between [+dist] and [−dist] UQs as allomorphy conditioned by the morphosyntactic number of the complement. But matters are more complicated. First, there is a tendency for [+dist] UQs to show more internal morphosyntactic complexity than [−dist] UQs, indicating that a structural difference rather than mere allomorphy is at work. Second, the distribution of the [+dist] forms seems to be conditioned by semantic properties of the complement extension, rather than morphosyntactic number.

We capture these facts by proposing that [+dist] UQs are structurally complex, containing an additional syntactic head one, which introduces a semantic restriction satisfied by singular count NP complements, but not by plural complements. We implement this within a realizational approach to morphology, in which vocabulary items can spell out a “span” of several heads in a functional sequence (see, e.g., Svenonius 2012; Taraldsen 2018; Blix 2021 for the notion of a span, Caha 2020; Baunaz et al. 2018 for complex spell-out more generally). From this perspective, [+dist] UQ forms like English *every* can be viewed as portmanteaus of $Q_{\forall}$ and one. Since the spell-out of $Q_{\forall}$ is sensitive to the presence or absence of one, it depends only indirectly on morphosyntactic number.

### Internally complex distributive quantifier forms

Our hypothesis that the difference between [+dist] and [−dist] UQs is not a case of allomorphy conditioned by number is based on two observations that are not straightforwardly compatible with such an account. The first one is that UQ forms in 2-form languages may be internally complex, and that *[*+*dist] UQs can be derived from [*−*dist] UQs*. This is illustrated in Table 3.

**Table 3:** Morphosyntactically complex [+dist] forms

language	source	[−dist]	[+dist]
Q’anjob’al (Mayan)	O’Flynn (2017)	*masanil*	*ju-jun*
		uq_1_	one-one
Hindi (IE, Indic)	Mahajan (2017)	*saar-ii/saar-e*	*praty-ek*
		uq_1_-pl	uq_2_-one
Western Armenian (IE, Armenian)	Khanjian (2012)	*amen*	*amen (meg)*
		uq	uq (one)
Georgian (South Caucasian)	Gil (1995)	*q’vela*	*q’ovelma*
		uq_1_	uq_2_
Kipsigis (Bantu)	Landman (2018)	*tugul*	*tugul age*
		uq	uq some

What stands out in Table 3 is that [+dist] forms often seem to involve the numeral “one.” Western Armenian as described in Khanjian (2012) even shows a transparent containment relation in which the [+dist] UQ form consists of the [−dist] form plus a morpheme formally equivalent to the numeral “one,” illustrated in (32) and (33).^15^ As shown in Table 3, Kipsigis also exhibits a transparent morphosyntactic containment relation between the [+dist] and the [−dist] form, but the additional morpheme included in the [+dist] form is analyzed as an indefinite article and not directly formally equivalent to the numeral “one” (Landman 2018).

(32)

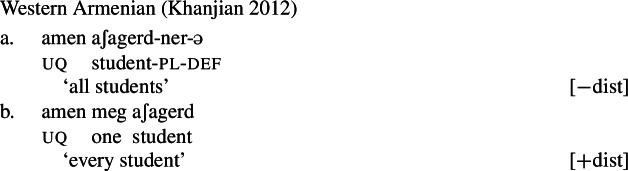

(33)
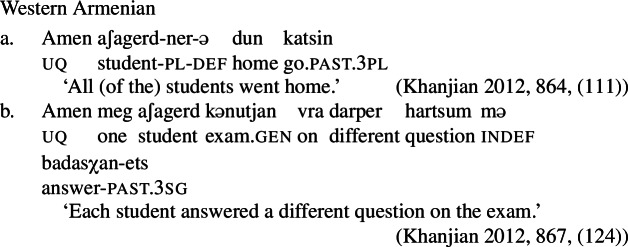

Such data suggest that the syntactic structure of [+dist] UQs should involve an element related to the numeral “one” that is absent in [−dist] UQs. Introducing an extra element in [+dist] structures would also fit well with less transparent cases: Gil (1995) suggests that the Georgian [+dist] form *q’ovelma* is derived from the [−dist] form *q’vela*, although not via a synchronically productive process.

Moreover, an account where the formal difference between [−dist] and [+dist] is determined exclusively by morphosyntactic number would not explain the second observation, namely, *semantics/morphology mismatches*, and thus would not fully derive the distribution (see also Fassi Fehri 2020).

While this issue will be discussed in more detail in Sect. 7, we mention the two crucial data points already at this point. First, in many languages at least some of the UQ forms found with count nouns can also combine with mass nouns. The UQ forms giving rise to a genuine mass interpretation (as opposed to quantification over portions or subkinds) are consistently [−dist] forms, even if the mass nouns bear singular inflection or trigger singular verb agreement, as illustrated for English in (34). This pattern is surprising on the view that [+dist] and [−dist] UQs are allomorphs conditioned by number, but expected if the choice between these forms is sensitive to the semantic property of being closed under sum, which plural count nouns share with mass nouns.

(34) All the water was gone. / *Every water was gone.

Second, several unrelated languages permit a non-distributive use of UQs with singular complements, on which the UQ contributes a similar meaning as English *whole*. As illustrated by the Greek example in (35), the UQ forms occurring in such constructions in 2-form languages are generally [−dist] forms. From a semantic perspective, this is unsurprising, as the UQ contributes maximization rather than distributivity. But if [−dist] forms were allomorphs conditioned by number, we would expect the [+dist] forms to surface in such examples.

(35)
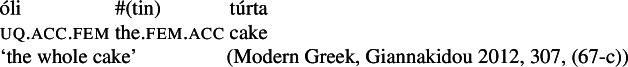

In Sect. 7, we argue that our approach to the semantics of UQs extends straightforwardly to these data. For now, the point is that in both of these cases, the choice between UQ forms seems to be conditioned by the semantics of the complement rather than its number morphology. We therefore propose an analytical approach in which the role of syntactic number is only indirect, and the tendency for [+dist] UQ forms to contain the numeral “one” is indicative of their internal structure.

### Background: Assumptions about realizational morphology

Before spelling out this approach, we briefly outline the morphosyntactic assumptions it makes use of.^16^

We employ a realizational morphological framework in which the morphology “interprets” the output of syntactic derivations rather than feeding them (as in Distributed Morphology, Halle and Marantz 1993), but assume, following the cartographic tradition, that features generally correspond to separate functional heads rather than being bundled in a single head (e.g., Kayne 2008; Cinque and Rizzi 2009). We further assume that a single exponent can directly serve as the realization of several syntactic heads, without a prior “fusion” operation of the kind assumed in *distributed morphology*. Vocabulary items spelling out multiple syntactic heads in one step are commonly associated with Nanosyntax (see, e.g., Starke 2009; Baunaz et al. 2018; Caha 2020), but the idea has been used by authors who do not adopt all the tenets of that framework (e.g., Abels and Muriungi 2008; Svenonius 2012, 2020; Blix 2021 a.o.), and also been explored within *distributed morphology* (Radkevich 2010; Bobaljik 2012).

Here we propose that the variation in whether or not [+dist] forms are transparently internally complex can be understood on what is often referred to as a *spanning* approach to complex spell-out (see, e.g., Abels and Muriungi 2008; Taraldsen 2010; Svenonius 2012; Taraldsen 2018; Blix 2021; several authors attribute the basic idea to Williams 2002 and note a close connection with Brody’s 2000 Mirror Theory). The core idea is that a vocabulary item can realize a “span” of heads—a contiguous subsequence of a functional sequence related by head-complement relations.

(36) A *span* is a finite sequence of syntactic heads $\langle X_{n}, \ldots , X_{1}\rangle $ such that, for 1 ≤ *i*<*n*, the maximal projection headed by $X_{i}$ is the complement of $X_{i+1}$.
  (adapted from Blix 2021)

Following Blix, we write [$X_{n}$ [$X_{n-1} \cdots $ [$X_{1}$]]] for the span $\langle X_{n}, \ldots , X_{1}\rangle $, without assuming that this span must form a constituent (i.e. $X_{1}$ could potentially take a complement that does not belong to the span). This means that in a highly restricted set of cases, vocabulary items can realize non-constituents. This differentiates the spanning view from the view generally assumed in Nanosyntax, on which vocabulary items have to match subtrees of a syntactic tree (for a comparison, see Taraldsen 2018, who concludes that the subtree view is superior).^17^

Here, we adopt the spanning view mainly for simplicity: It prevents us from having to represent and motivate the various movement operations assumed in Nanosyntax to create subtrees matching the complex syntactic objects stored in the lexicon. Specifically, we will not assume that the spell-out algorithm triggers movement or last-resort insertion of features (cf. Caha et al. 2019; Taraldsen 2018), and more generally leave open whether the surface linearization of the items spelling out different spans is derived by syntactic movement.

Unlike in Distributed Morphology, where a vocabulary item must have a subset of the features of the head at which it is inserted, both Nanosyntax and some versions of the spanning approach assume that vocabulary insertion is governed by a Superset Principle: A vocabulary item must have all the features of the chunk of structure it spells out. This permits the insertion of vocabulary items that have superfluous features, but not insertion of a vocabulary item that lacks a feature present in the syntactic span it is supposed to realize. For concreteness, consider the following condition on matching between a vocabulary item and a syntactic span (Abels and Muriungi 2008):

(37) A vocabulary item that lexicalizes a span $\langle X_{n}, \ldots X_{1} \rangle $ matches a syntactic span *S* iff there is a *m* ≤ *n* such that $S = \langle X_{m}, \ldots X_{1}\rangle $.
  (adapted from Blix 2021)

Note that (37) requires the matching syntactic subspan to reach all the way down to the lowest feature $X_{1}$, a condition that will be weakened somewhat later on. The choice between multiple matching vocabulary items is assumed to be regulated by a version of the Elsewhere Condition that favors vocabulary items with fewer unnecessary features.

### The structures underlying [+dist] and [−dist] UQ

We will draw on this background once we have established the basics of the analysis: We posit a functional element one right below $Q_{\forall}$, which has a semantics closely related to the numeral “one” and which is not present in the structure corresponding to [−dist] UQs. This hypothesis is illustrated in (38) and (39). (We assume that any relevant *ϕ*-features other than number are encoded as functional heads below #. As their exact representation does not matter here, we represent them as a single head F.)

(38)

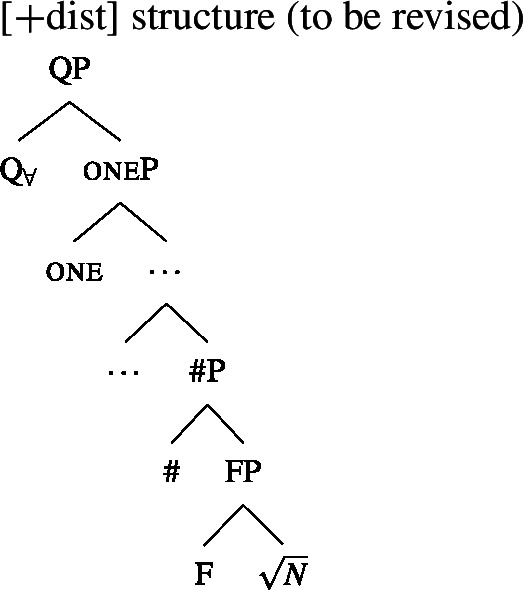

(39)
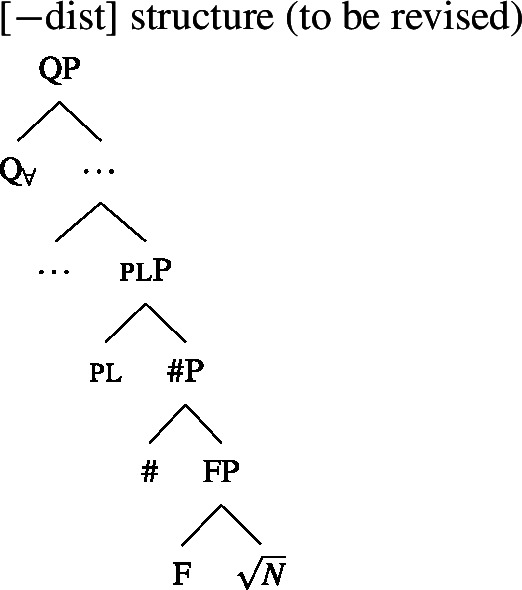

To be able to derive the DNG from these structures, we need two restrictions on the distribution of one: (i) one is blocked from co-occurring with plural NP or DP complements, and (ii) one must occur whenever $Q_{\forall}$ combines with a singular NP.

Restriction (i) can be derived in the semantics. We assume that one adds the presupposition that the complement’s extension contains at least two individuals and no two of them overlap. The “at least two” condition prevents “accidental” licensing of [+dist] UQ forms in the case where the noun extension happens to be a singleton. While (40) differs from the standard lexical entry for the numeral *one*, which removes overlap from a predicate extension by picking out the atomic individuals rather than presupposing non-overlap, it seems close enough for there to be a plausible grammaticalization path from the intersective to the presuppositional meaning.^18^ (See Sect. 6 for a refinement of these assumptions about one and a discussion of semantic variation among [+dist] UQs.)

(40) 〚one$]\!\!]{}=\lambda P_{\langle{}\alpha , t\rangle{}}$ : $|P| > 1 \land \forall x_{\alpha}$, $y_{\alpha} \in P[x \neq y \rightarrow \neg \exists z[z \sqsubseteq x \land z \sqsubseteq y]]. P$

To see why it is impossible to combine one with a plural complement, consider the trees in (41) and (42). In (41), the complement of one is a #P, which we assumed denotes a set of atomic individuals, so the non-overlap presupposition is met. But in (42), the pl head below one guarantees the presence of plural individuals in the extension. Each of these overlaps with some atomic individuals, so the presupposition of one is violated whenever pl is present. In Sect. 6, we will introduce a semantics for plural DPs on which the co-occurrence of one with a plural DP is blocked along the same lines.

(41)
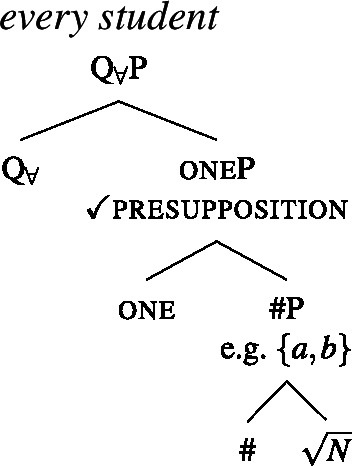

(42)
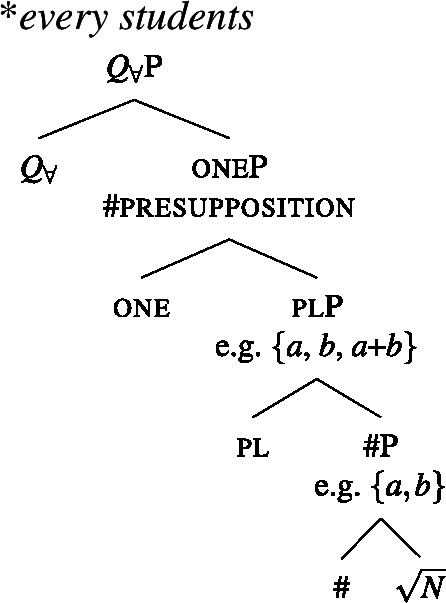

The source of restriction (ii) is less obvious. One option would be to stipulate a restriction on the minimum size of the complement of $Q_{\forall}$ (cf. Wurmbrand and Lohninger 2023 for similar ideas in a different empirical domain): at least in 2-form languages, $Q_{\forall}$ is restricted from merging with a complement as small as a #P. This restriction could be circumvented by making the complement plural as in (39) or making the complement a DP, but given our semantics, neither option results in a [+dist] UQ interpretation: A plural complement yields a [−dist] UQ and a DP complement yields a “whole” meaning. Inserting one would then be the only way of meeting the restriction that gives rise to a [+dist] semantics.

Another analytical option we want to raise, without exploring it in detail, is to appeal to a pragmatic principle to force the insertion of one. Since we analyzed one as a presupposition trigger, this principle could be a generalized version of *Maximize Presupposition!* (Heim 1991; Sauerland 2008a; Percus 2006 a.o.). Given an expression *ϕ* and a contextually equivalent alternative *ψ* with a stronger presupposition, *Maximize Presupposition!* requires the use of *ψ* if the presuppositions of both alternatives are met. The standard version of the principle requires the presuppositionally stronger alternative *ψ* to be no more complex than *ϕ*, and thus would not force us to use *ψ*= [one [# $\sqrt{N}$]] rather than *ϕ*= [# $\sqrt{N}$]. However, there are other phenomena that have been analyzed in terms of *MP!* and, on a “cartographic” approach to *ϕ*-features, involve similar complexity asymmetries. For instance, third person on pronouns has been argued to be presupposition-less and receive its semantic content by means of competition with local person features under *MP!* (see, e.g., Sauerland 2008b), but if person features are analyzed as separate heads rather than components of a feature bundle, local person pronouns end up being syntactically more complex than third person pronouns. The question arises whether one could define a generalized version of *MP!* that applies in such cases without forcing the insertion of arbitrary amounts of structure in order to strengthen the presupposition (cf. Sauerland 2008a for cases in which more complex alternatives are *not* available for *MP!*). If this is feasible, it would provide a purely semantic/pragmatic way of forcing one to be inserted that does not rely on a stipulative complement-size restriction.

In sum, we have given one a semantics that makes it incompatible with plural complements, and informally sketched two ways of forcing its presence with singular complements—a stipulative selectional restriction or a pragmatic competition mechanism favoring stronger presuppositions that is more general than usually assumed.

Whatever the right means of capturing the distribution of one, its presence provides a new account of the different realizations of $Q_{\forall}$ in 2-form languages: The [+dist] forms realize both $Q_{\forall}$ and one, while the [−dist] forms are the realizations of $Q_{\forall}$ selected in the absence of one. This idea is compatible with the two properties listed in Sect. 5.1: (i) the observation that [+dist] forms are often morphologically complex and tend to contain the numeral *one* is naturally accounted for; and (ii) we expect to find [−dist] forms with morphologically singular complements, as long as there is a reason for these complements to lack one. We will return to the latter issue in Sect. 7.

The idea that the structures of certain distributive UQs contain an element related to the numeral *one* is not new: Zimmermann (2011) argues that Low German provides evidence for an additional functional layer NumP within the DP, which surfaces in pronominal quantifiers as the suffix *-een* (“one”) (e.g., *jeder-een* ‘every-one’, or *keen-een* ‘no-one’). Zimmermann (2011) notes that the *-een*-suffix is restricted to UQs taking singular count NP complements, but never attaches to UQs with plural and mass complements. He thus suggests that in the structures of UQs with singular complements, the NumP carries a syntactic [+singular] feature that must agree with a corresponding feature on the (potentially covert) NP, triggering the presupposition that the NP-denotation consists only of atomic individuals. In contrast, in the structures of UQs with plural/mass complements, NumP carries a [−singular] feature.

That the numeral “one” plays a role in the decomposition of distributive UQs is also proposed in Jayaseelan (2011), based on the morphology of distributive quantifiers in Malayalam (43).^19^

(43)
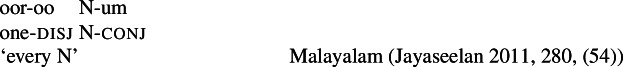

Fassi Fehri (2020) proposes another variant of a “one” element for a subset of distributive UQs, motivated by data from Semitic languages. However, he takes this element to be a pro-N in a partitive structure and assumes it is absent in [+dist] UQ forms that do not combine with partitives, such as *every*. We will return to his proposal in Sect. 6.2, where we discuss variation among [+dist] UQ forms, and in Sect. 7.3, where we compare his purely syntactic account of [+dist] forms to our semantic approach.

We now turn to the consequences of our new structural proposal for the postsyntactic vocabularies of different 1-form and 2-form languages.

### Deriving the surface patterns

Our point of departure are the underlying syntactic structures we assume for [+dist]/[−dist] UQs in (38)/(39).

We assume that the complement of oneP in the singular case, and $Q_{\forall}P$ in the plural case, is opaque to the spell-out mechanism, either because it has already received its final realization or because it has undergone movement to create a constituent corresponding to the UQ. We remain neutral about this choice and indicate this by writing the category label of the complement in gray as in (44)/(45).

(44)

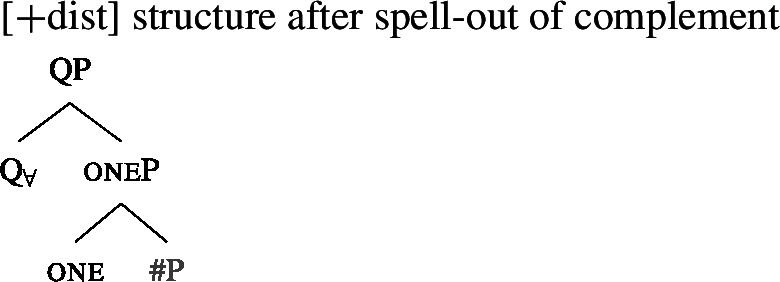

(45)
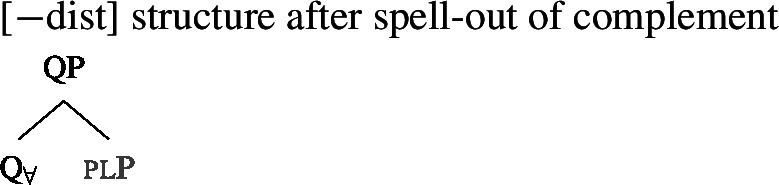

We start with 1-form languages like Dagara, where there is no morphological evidence for the presence of the head one. In principle, we could assume that one is absent in such languages. But we could also take one to have a null realization, which would permit keeping the uniform structure in (44) for both 1-form and 2-form languages. In this case, the relevant part of the lexicon of Dagara would then be as in (46b,c). (As we currently have no means to decide between these two options, we are not committed to either.)

(46)
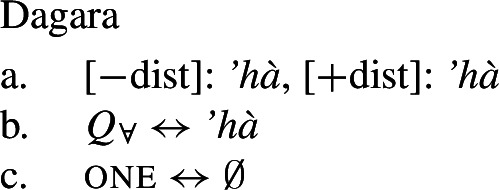

Given (46), we might expect to find languages that have the same pattern, except that the phonological realization of one is overt. This case is represented by Western Armenian, where a [+dist] UQ can be formed by combining the [−dist] form with the numeral *one*:

(47)
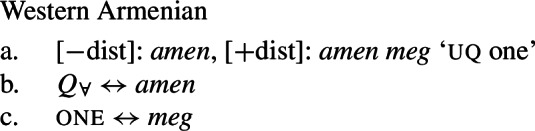

Returning to the English pattern, where neither of the two UQ forms is (synchronically) internally complex, we can still account for the contrast in terms of the vocabulary items in (48). Since (48c) does not contain the feature one, (48b) is now the only vocabulary item that matches the structure in (44) once the complement of oneP has been spelled out. In the structure (45), (48c) must be selected since the lowest feature in the span lexicalized in (48b), one, does not appear in the tree.^20^

(48)

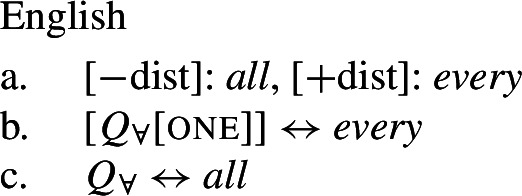

### $Q_{\forall}$ and number agreement

We now have a way of deriving the pattern in 2-form languages that is consistent with the two observations from Sect. 5.1, namely (i) that [+dist] UQs can be internally complex and (ii) that their distribution seems to be governed by a semantic property rather than syntactic number. These observations are derived from a decomposition of [+dist] UQs that is only indirectly related to number, via the semantics of one. This gives the proposal an advantage over an alternative view that would also be compatible with the Single Quantifier Meaning Hypothesis and the spanning approach—that the [+dist]/[−dist] distinction is reducible to syntactic number agreement between the UQ and its complement.

To illustrate the latter approach and its problems, we need to say something about the syntactic structure underlying number agreement on UQ forms. Within the framework assumed here, which eschews feature bundles and treats features as separate heads whenever possible, feature agreement presumably has to be implemented as a form of countercyclic structure insertion (see Deal 2025 for an argument supporting this view of agreement). Here, we will therefore assume that in languages showing number agreement on UQs, the number projections associated with the complement NP/DP are repeated above the quantifier at the level that forms the input to spell-out. Given this assumption, one might attempt to analyze the [+dist] and [−dist] forms as spelling out a span consisting of $Q_{\forall}$ and number, as in (49) and (50).^21^

(49)

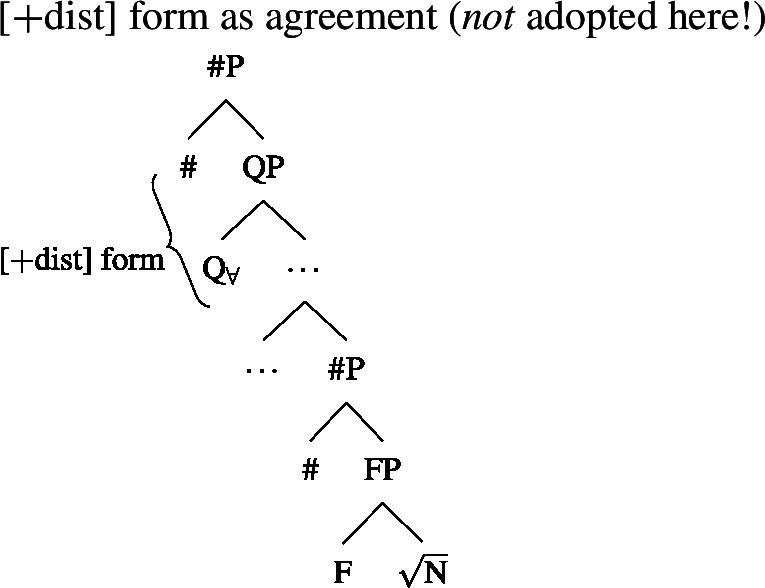

(50)

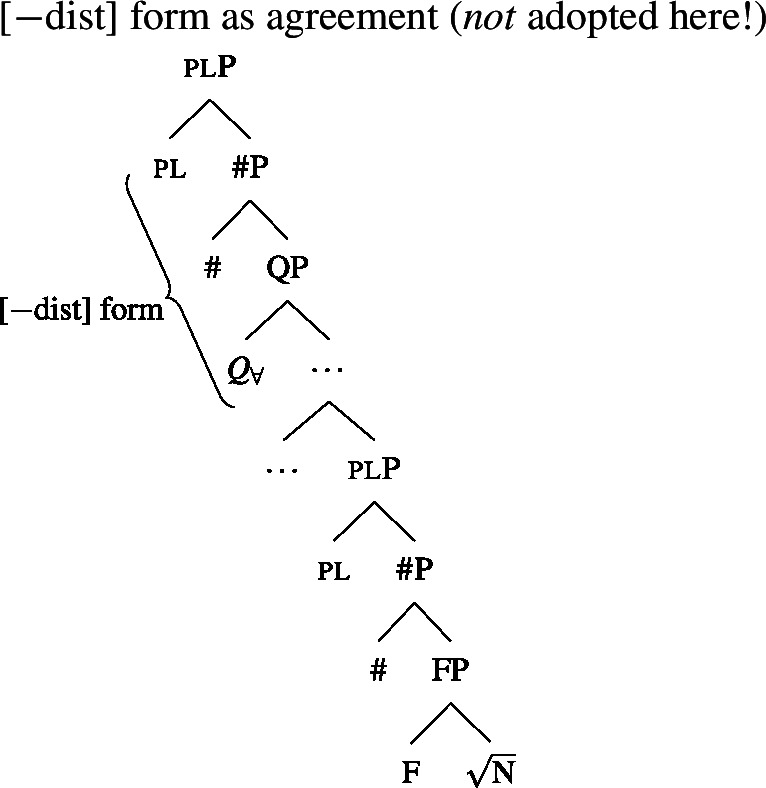

The vocabulary items that would be necessary to implement this approach are illustrated in (51). In (49), both items would match the span from $Q_{\forall}$ to #, but the Elsewhere Condition would favor (51a), which lacks the unnecessary pl feature of (51b). In contrast, in (50), only (51b) would be a possible match that realizes all the features from $Q_{\forall}$ upwards.

(51)
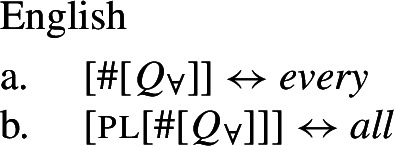

While seemingly intuitive, such an approach would lead us to expect any internal morphosyntactic complexity in [+dist] forms to be shared with [−dist] forms, since the latter would spell out a strictly more complex chunk of the tree. As discussed in Sect. 5.3 above, this expectation is not borne out. Further, this proposal provides no room for mismatches between number and the distribution of the [+dist]/[−dist] forms, to be discussed in more detail in Sect. 7. Therefore, the analysis in terms of one proposed in the previous section outperforms an alternative approach based on number agreement.

That being said, even on our approach there is some motivation for agreement projections above $Q_{\forall}$ (on a language-specific basis), since any adequate theory has to account for the fact that UQ forms can show *overt number agreement* with the complement NP or DP. In line with the DNG, this agreement is singular in [+dist] UQs and plural for [−dist] UQs, as illustrated for German in (52).

(52)
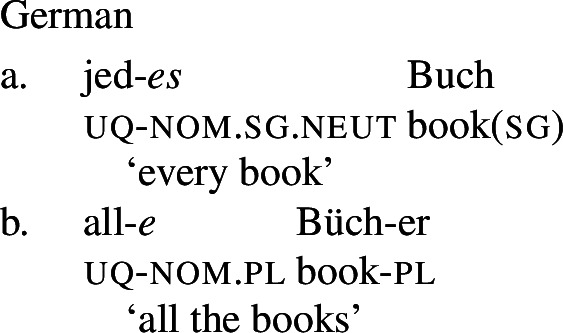

In such languages, the relevant part of the functional sequence could be divided into two spans: First, the UQ element itself, which comes out as *jed*- if one is present and *all-* otherwise (53b-c), and second, the agreement marker (53d-e). (The lexical entries in (53) are of course oversimplified and gloss over case and gender agreement.) In the singular case, (53e) must be selected due to the Superset Principle; in the plural case, both vocabulary items match, but (53d) must be selected due to the Elsewhere Condition. The vocabulary items for the agreement markers do not contain any features specific to UQs, which reflects the fact that exactly the same markers occur in the so-called strong agreement paradigm of non-quantificational adjectives (see Leu 2009 for discussion^22^).^23^

(53)
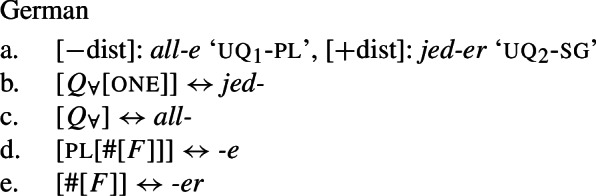

The trees in (54) and (55) illustrate the parts of the functional sequence realized by each marker in German. We remain non-committal on the issue of what ensures that the agreement markers are suffixed to the UQ forms rather than preceding them, as is the case in general for German adjectives. One option would be to posit movement of the QP to the specifier of the highest number projection, which would then create a constituent corresponding to the agreement marker that must be linearized to the right (see Leu 2009 for a movement-based approach to the distribution of German “strong” adjective agreement more generally).^24^ This option is schematized in (56) and (57). One open question for such proposals, however, is how to ensure in a principled way that the complements of oneP in (56) and of $Q_{\forall}$P in (57) move out so that they are linearized to the right of the agreement markers and do not form a surface constituent with the UQ, and that the same consistently happens for other elements in the extended NP that bear suffixal agreement.^25^ Alternatively, some of the literature making use of spanning has proposed linearization mechanisms that do not rely on movement (see, e.g., Svenonius 2020). Since our main concern in this paper is morphosemantics, we leave the matter open here.

(54)

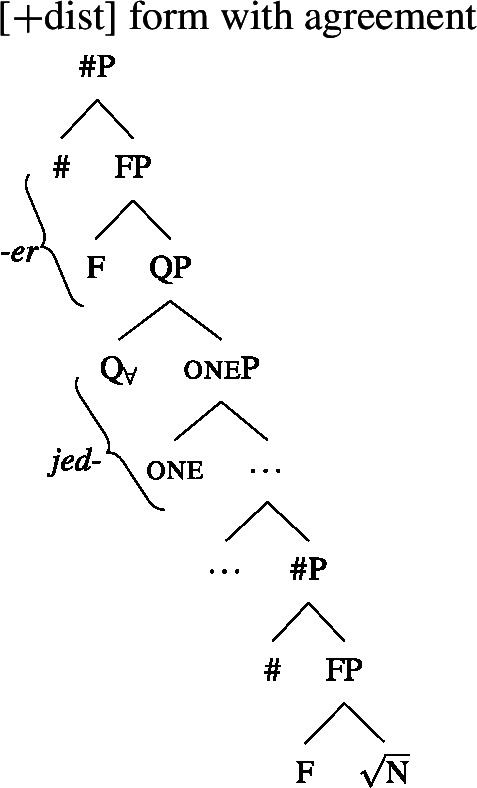

(55)

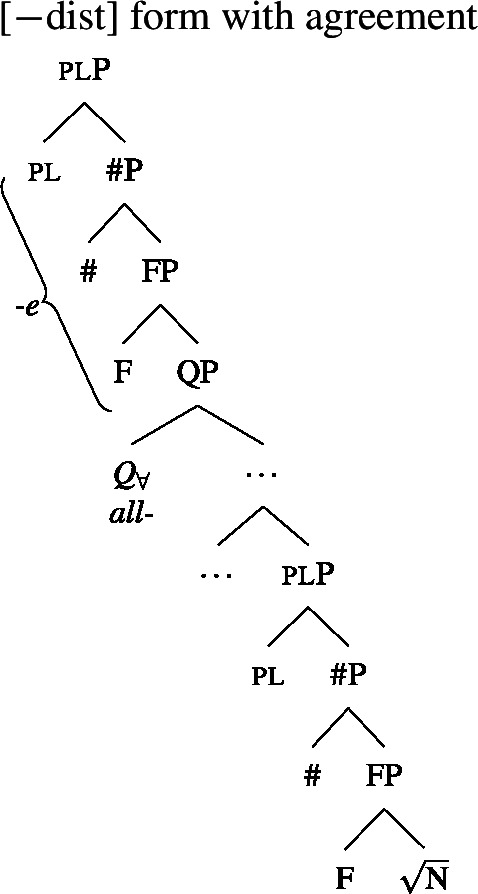

(56)

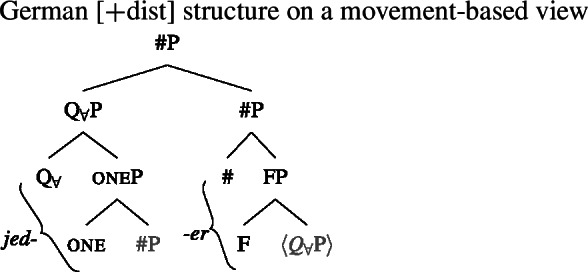

(57)

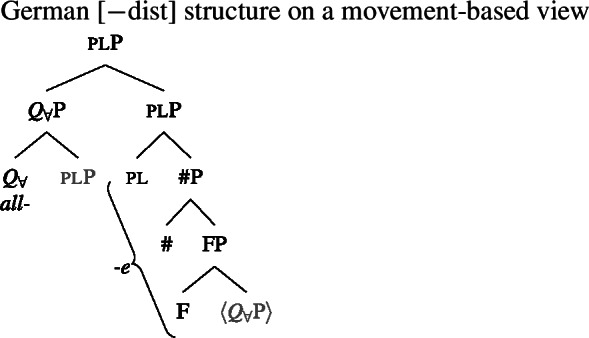

In sum, while there are some open issues (notably how agreement suffixes are linearized), we now have a way of deriving a wide range of attested UQ paradigms from a uniform underlying syntactic structure. The proposal captures several morphosyntactic properties of UQs: First, the additional primitive one in the structure of [+dist] UQs, introduced in Sect. 5.3, accounts for the tendency for [+dist] UQs to be internally complex. Second, since [+dist] forms are the result of joint spell-out of $Q_{\forall}$ and one (Sect. 5.4), they are not directly conditioned by number, which leads us to expect mismatches between the distribution of [−dist] forms and of singular number. Third, overt number agreement on UQ forms in 2-form languages is expected on our proposal since we take number agreement to be separate from the [+dist]/[−dist] distinction (Sect. 5.5).

At this point, an interesting consequence of the use of spanning to derive the different UQ forms is worth pointing out. Some languages have UQ strategies that differ in whether or not they combine with overt agreement marking. This is illustrated in (58) for Hindi, which has both a transparent realization of one in some of its [+dist] UQs and suffixal number agreement in some of its [−dist] UQs.^26^

(58)
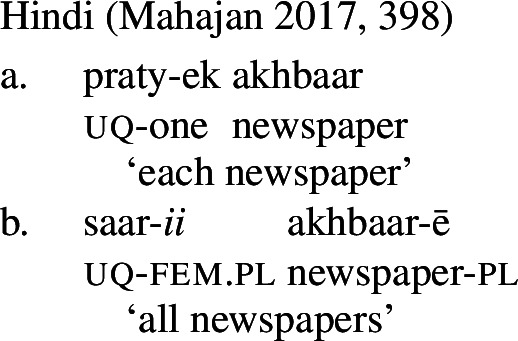

Two facts about these forms are interesting: (i) the lack of singular number agreement in the [+dist] form (note that the [−dist] exponent can combine with singular agreement in structures with a “whole” reading; see (59)) and (ii) the fact that there is a separate [+dist] $Q_{\forall}$ form, even though $Q_{\forall}$ and one cannot have a joint spell-out, since one is realized separately.

(59)
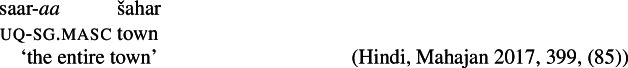

We tentatively suggest that both facts could be captured by assuming that in the [+dist] form, the $Q_{\forall}$ head and the *ϕ*-features constitute a span spelled out by a single marker, to the exclusion of one, as illustrated in (60).

(60)
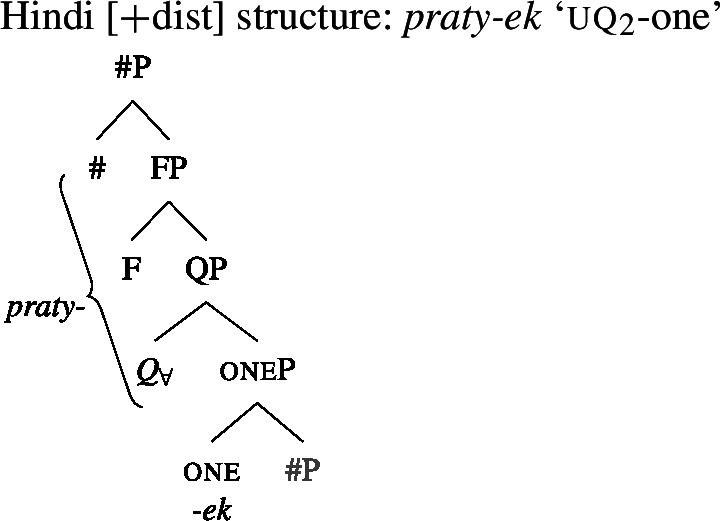

(61)
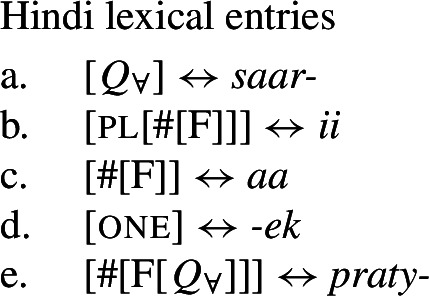

If so, however, the question arises how the form in (59) is derived: Assuming that the underlying structure is the same as in German (55), why don’t we have to realize the span [#[F[$Q_{\forall}$]]] as *praty-* in this case as well? Later on, we will independently motivate two ideas that, taken together, will resolve this puzzle: 1) that ‘whole’ readings of UQ with singular nouns as in (59) involve DP rather than NP complements (Sect. 7.2), and 2) that [−dist] exponents in some languages are underspecified between the spans [$Q_{\forall}$] and [$Q_{\forall}$[D]] (Sect. 6.4). Given these assumptions, the need to realize the D head can be used to force the [−dist] exponent to occur in examples like (59).

In sum, while the [+dist]/[−dist] contrast itself is not reducible to number agreement, UQ strategies within a language can differ in whether or not they co-occur with overt number agreement. This possibility is to be expected on a spanning approach, on which the $Q_{\forall}$ head and its associated number agreement can be spelled out by a single item.

### Partitives

Since we assume that the realization of UQs in 2-form languages is conditioned by the structure of their complements, the question arises why both forms are possible in partitive constructions, exemplified for German in (62):

(62)
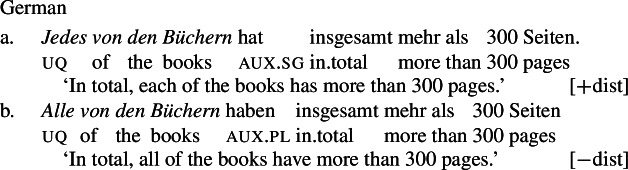

One possibility to account for these facts within the current proposal would be to assume that partitive constructions contain a silent pro-N, as suggested by Jackendoff (1977), Sauerland and Yatsushiro (2004). Sauerland and Yatsushiro (2004) propose that this pro-N, semantically, can either be an exact copy of the $\sqrt{\text{N}}$ embedded in the partitive phrase (i.e., $\sqrt{\textsc{book}}$ in (62)) or a semantically bleached predicate like that expressed by *thing*. Given such a silent pro-N, the (non-)distributivity and the spell-out of $Q_{\forall}$ are both predicted to be determined by its number features, and should therefore still correlate.^27^

Building on this idea, a possible structure for a singular partitive is (63), where the functional layers above N are the same as before (we omit the functional structure above QP). The presence of the # head licenses both the occurrence of one in the syntax and restricts the extension of the node dominating N_PRO_ and the partitive phrase to atomic individuals, so that one is semantically licensed.

(63)
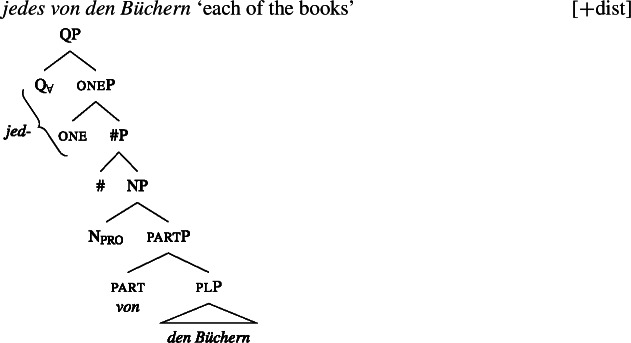

In (64) on the other hand, $Q_{\forall}$ combines with a plP that denotes a set of both atomic and plural individuals, and therefore ends up applying the nuclear-scope predicate to the maximal element of that set:

(64)

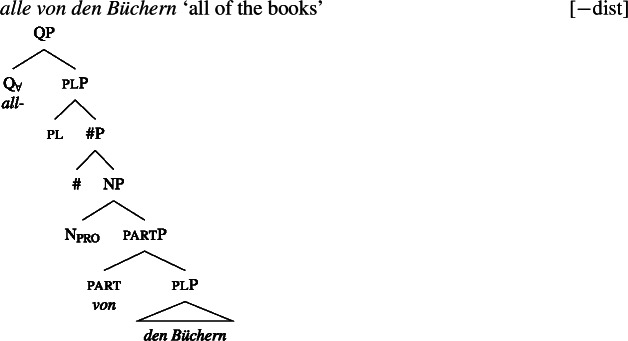

For the moment, we assume the standard semantics in (65) for the partitive marker: it takes a complement of type *e* (e.g., the sum of all the books in (63) and (64)), and returns a function that maps all and only the parts of this individual to true.^28^ The result is a predicate true of both atomic and plural individuals. (However, we will revise our account of the semantics of partitives in the next section, once we have adapted the semantics of DPs to allow them to combine directly with $Q_{\forall}$.)

(65) 〚part$]\!\!]{}=\lambda x_{e}. \lambda y_{e}. y \sqsubseteq x$ to be revised

The predicate returned by (65) can then be intersected with the silent N_PRO_. If the latter is semantically identical to $\sqrt{\text{N}}$ within the partitive phrase (i.e., $\sqrt{ \textsc{book}}$ in (63) and (64)), the intersection will leave the initial set provided by the partitive phrase intact (assuming $\sqrt{\text{N}}$ is not specified for number). Adding # on top of the resulting structure yields a set of atomic individuals (atomic boys in (63) and (64)). This licenses insertion of one in (63); adding $Q_{\forall}$ gives us the sub-structure spelled out as *jed-* and a [+dist] interpretation. But if we add pl instead of one, we derive the form *all-* and a [−dist] interpretation.

Note, however, that this is only one of the paths we could pursue—from a semantic perspective, the silent pro-N is not required, as the same semantic effect could be obtained by simply building the functional structure we assumed on top of N_pro_ directly on top of the partitive marker.

## Refining the proposal: Non-overlap, subtypes of [+dist] UQs and DP complements

We now address three issues that force us to refine our semantic and morphosyntactic proposal. The first issue is that our current version overgenerates unattested meanings for $Q_{\forall}$ in combination with numeral-modified indefinites. The second issue is that our current structural proposal does not capture the variation between [+dist] UQ meanings within and across languages. The third issue concerns $Q_{\forall}$ in the context of plural DPs, where we are a) confronted with a type-mismatch, and b) must make certain assumptions about the semantics of plural DPs to account for the maximality effects triggered by $Q_{\forall}$. After extending our account to UQ with plural DPs, we raise the question of why it is so common cross-linguistically for [−dist] UQs to take DP rather than NP complements, and sketch one potential answer. The semantics of UQ with singular DP complements will be addressed in Sect. 7 below.

### Numeral-modified indefinites

**Fig. 3:**
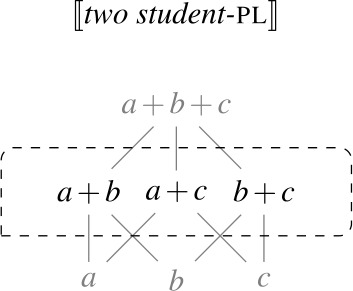
Maximal elements of numeral-modified plural NP extensions

Our current semantics for $Q_{\forall}$ predicts that when the complement of a UQ is modified by a numeral ≥2, the UQ can distribute down to the minimal pluralities meeting the size requirement of the numeral. Hence, when $Q_{\forall}$ combines with the denotation of *two students*, a set containing pluralities consisting of two atomic elements, the scope predicate is applied to each of these pluralities, as illustrated in Figure 3.

(66)

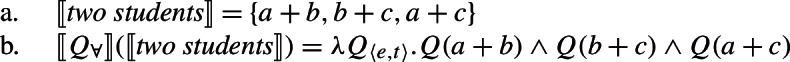

In the 2-form-languages English and German, the [−dist] form *alle/all* can combine with a numeral-modified complement. However, the expressed meaning is not the one predicted by (66b): while (67) is grammatical, it is only acceptable if there are only three salient boys in total, i.e., (67) cannot receive a meaning along the lines of (66-b), under which the predicate is required to hold of every plurality of three salient boys.

(67)
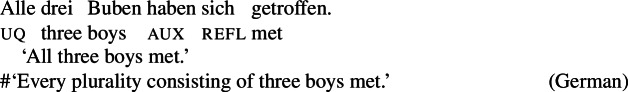

The same holds for Dagara, (68), and Wolof, (69), where $Q_{\forall}$ with a numeral modified plural complement yields the same interpretation.

(68)

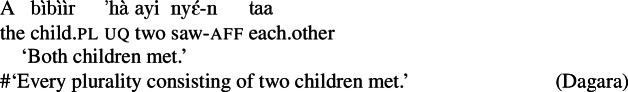

(69)

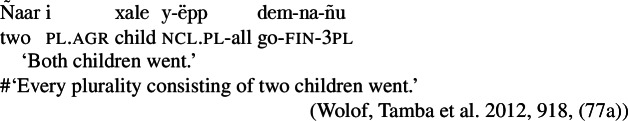

Intuitively, this suggests that $\mathcal{Q}_{\forall }$ should not quantify over all maximal elements of the restriction: If there are several maximal elements that overlap, these overlapping elements should be excluded from the domain of $\mathcal{Q}_{\forall }$. We implement this requirement by revising our semantics for $\mathcal{Q}_{\forall }$ as in (70). The intuition in (70) is that the predicate applies only to those elements of its restrictor set that do not overlap with any other elements of the restrictor set, except for their parts. (Again, we use *α* as a variable over different types for which a part-whole structure is defined.)

(70)
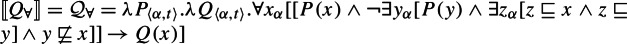

Combining this updated quantifier denotation with the denotation usually assumed for *two students* correctly rules out the meaning in (66-b): As every maximal element in the denotation (66-a) overlaps with another maximal element, $Q_{\forall}$ now quantifies vacuously over the empty set. So for $Q_{\forall}$ to be able to combine with *two students*, the extension of *two students* must be a singleton set containing a single plurality of two students, i.e., there must be exactly two salient students.

Note, however, that this account does not make it impossible for $Q_{\forall}$ to combine with a non-singleton set of pluralities: If a contextual domain restriction mechanism (e.g., restriction to a “cover” in the sense of Schwarzschild 1996) could remove the overlap from a set of pluralities, then $Q_{\forall}$ would be predicted to be acceptable with plural complements. But crucially, the result would not be a standard distributive reading, but distributive quantification over a set of non-overlapping salient “subgroups.”

In the languages mentioned above, this reading is not available, so we conclude that restriction by means of covers is not possible before the quantifier is merged. However, this property might not be universal. Landman (2016) reports that in Logoori, the [+dist] UQ form *vuri* can combine with a plural complement, resulting in a reading that obligatorily involves distribution over subgroups; Roni Katzir (p.c.) informs us that the same is possible for Hebrew *kol* with a numeral-modified complement. We speculate that these cases might involve cover-based domain restriction, resulting in a domain without overlap, but more work is needed to see if this suggestion is tenable.

Having fixed the first issue by revising the semantics for $Q_{\forall}$, we now turn to another complication concerning numeral-modified complements.

### Subtypes of [+dist] UQs and atomicity

We have just seen that in several languages, including English, German, Dagara, and Wolof, UQ forms combining with a numeral-modified plural NP express maximality, rather than distributive quantification. However, there is a systematic set of counterexamples to this claim, involving measure phrases:

(71)
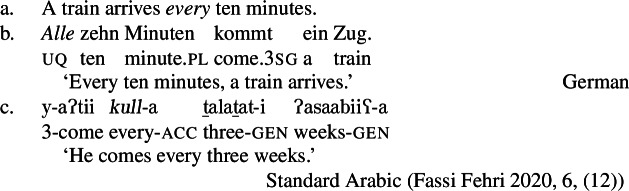

Semantically, these sentences can be accommodated in our system, given two assumptions: First, we must extend the part-whole relation ⊑ to degree intervals (where *x*⊑*y* means that the interval *x* is included in *y*; for reasons of space, we omit the formal details). Second, we must assume that a measure predicate like *ten minutes* can be coerced into an interpretation on which it is true of non-overlapping intervals of ten minutes, as illustrated in (72). If so, these non-overlapping intervals are all maximal within the extension of 〚*ten minutes*〛, permitting $\mathcal{Q}_{\forall }$ to range over all of them.

(72) 〚*ten minutes*〛 = {…[9:50am,10:00am),[10:00am,10:10am),[10:10am,10:20am),…}

But we still face a problem concerning the UQ *forms* used in such constructions. Our account predicts that all 2-form languages should permit [+dist] forms with degree-interval predicates like (72). However, some [+dist] UQ forms are incompatible with such predicates, as illustrated in (73) and (74) (the oddness of (74-b) is subject to variation).

(73)

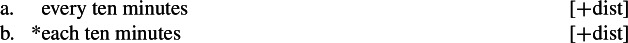

(74)
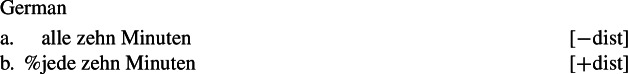

Following Fassi Fehri (2020), we take this to be indicative of an additional parameter of variation between [+dist] UQs: We suggest that the reason why *every* is compatible with degree-interval predicates like *ten minutes* and *each/jeder* are not is that *every* merely requires non-overlap between the individuals satisfying its restrictor predicate, while *jeder* and *each* impose the stronger condition of *atomicity*: A [+dist] UQ form like *jeder* or *each* requires its restrictor predicate to be true only of entities that are not sums of two or more distinct parts. (We will have to revise this condition somewhat once we discuss UQ ranging over mass and “subatomic” parts.) It is plausible to assume that a predicate of degree intervals cannot satisfy this requirement, as an interval (of, e.g., ten minutes) can always be expressed as the sum of shorter intervals. We descriptively refer to UQs of the *each/jeder* type that do not combine with degree-interval predicates as [+atomic] and to UQs of the *every* type that do combine with such predicates as [−atomic].^29^

Given our Single Quantifier Meaning Hypothesis, the source of this variation between UQ forms should not be tied to the quantificational element $\mathcal{Q}_{\forall }$, but to the structural primitives it combines with. We propose that there are two such primitives. One is our element one from above; we rename it one_∅_ to indicate its non-overlap requirement (75a). The second primitive, one_at_, requires the predicate it combines with to be true of atomic entities only (75b) (we write $AT_{\alpha}$ for the atomic elements of the semantic domain $D_{\alpha}$). It will thus yield a presupposition failure with a restrictor predicate like *ten minutes*.

(75)
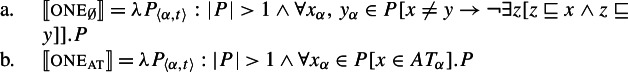

What do the structures of [+atomic] distributive UQ forms look like? The simplest hypothesis would be that they are identical to the [−atomic] forms, except for the lexical choice between the two one-elements. We suggest instead, following Fassi Fehri (2020) (but deviating from the details of his proposal) that in languages distinguishing between [+atomic] and [−atomic] UQ forms, the former are structurally more complex (cf. also Jayaseelan 2011 on ‘each’). We implement this by assigning them a functional sequence with one_∅_ on top of one_at_—so languages like English, which distinguish two [+dist] UQ forms, have the three structures in (76). This will descriptively capture differences in the selection patterns of [+dist] UQ forms; for example, *every* cannot take partitive complements but *each* can.

(76)
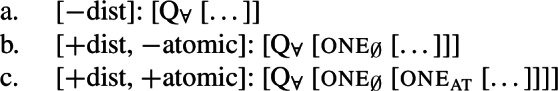

While all three UQ structures are possible with count predicates whose extensions are based on atoms, degree-interval predicates like *ten minutes* are semantically blocked from appearing in the configuration (76c). However, the structures in (77a) and (77b) are both semantically licensed.

(77)
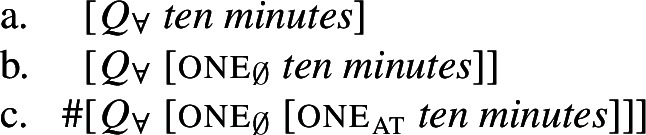

Descriptively, some languages, such as English, require one_∅_ with degree-interval predicates, as in (77b), while other languages, such as German, do not. We do not fully understand the source of this variation, but speculate that German patterns with 1-form languages in lacking one_∅_ and therefore permits only the following two UQ structures:

(78)
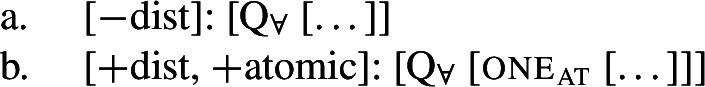

The resulting proposal allows us to capture the semantic variation between UQ systems in a more fine-grained way. For English, we only need to add the lexical entry in (79c) for *each* to capture the contrast between *every* and *each*. This derives the contrast between *every ten minutes* and *each ten minutes.

(79)
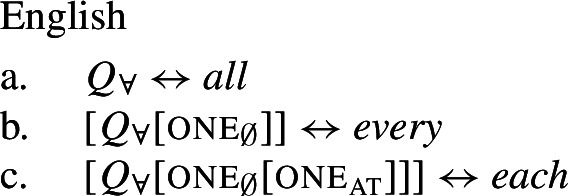

To derive the incompatibility of German *jed-* with degree-interval predicates, we assume the vocabulary items in (80). Since we assumed that German lacks one_∅_ in the functional sequence,^30^ and since one_at_ cannot combine with degree-interval predicates, the only licit structure for UQ with a degree-interval predicate in German is (77-a), resulting in the form *alle zehn Minuten*.^31^

(80)
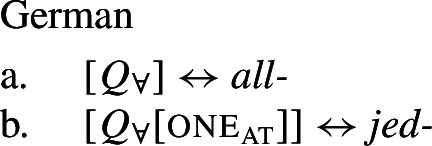

A [+atomic] distributive UQ structure is also found in Arabic, a 1-form language. Fassi Fehri (2020) shows that the UQ form *kull* in Standard Arabic, which is underspecified between [+dist] and [−dist] meanings, can be combined with the numeral *waaḥid* ‘one’ and that this numeral adds an atomicity requirement (among other semantic effects). As (71-c) shows, *kull* itself, without the numeral, does not require atomicity. We can now account for this by proposing 1) that Arabic, like German, lacks $\textsc{one}_{\emptyset}$, and 2) that *waaḥid* is the spell-out of one_at_.

(81)
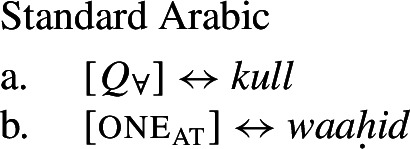

French exhibits a related pattern: it uses the form *tout/toutes/tous* for both [−dist] and [+dist] UQ, but has the additional [+dist] form *chaque*. Corblin (2018) and Fassi Fehri (2020) suggest that *chaque* requires atomicity, while *tout* (even on its singular, [+dist] use) does not.^32^ If so, *chaque* will spell out a structure with both one-elements, while *tou-* can spell out either of the two smaller UQ structures $[Q_{\forall} [\textsc{one}_{\emptyset}]]$ and $[Q_{\forall}]$.

Technically, the system employed so far prohibits introducing a vocabulary item that matches both $[Q_{\forall} [\textsc{one}_{\emptyset}]]$ and $[Q_{\forall}]$, as the lowest feature in the vocabulary item must have a match in the syntactic tree. We therefore propose to expand the expressive power of the spell-out mechanism by adding *pointers*, a formal tool that was originally introduced in the Nanosyntax literature (Caha and Pantcheva 2012; Caha et al. 2019 a.o.), but adapted for a spanning approach to spell-out in Blix (2021).

In Blix’s system,^33^ a lexical entry with a pointer, written as $[S_{1} \rightarrow [S_{2}]]$ (where $S_{1}$ and $S_{2}$ are two spans), matches any nonempty part of the functional sequence that consists of a subspan matching $S_{1}$ followed by a subspan matching $S_{2}$. These subspans can be empty, so in the absence of any features from $S_{2}$, a match with $S_{1}$ is sufficient. With this formal tool, the French UQ system can be analyzed as follows, where (82a) matches the syntactic spans [$Q_{\forall}$] and [$Q_{\forall}$[one_∅_]].

(82)
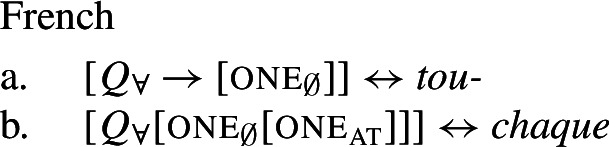

In sum, we observed that many languages have several [+dist] UQ forms that differ in the strictness of the requirements on their complements. We can integrate this into our proposal if we posit multiple elements that can attach to the complement of $Q_{\forall}$ in [+dist] structures, at least on a language-specific basis if not universally.^34^

### Plural DPs

So far, we only discussed the behavior of $Q_{\forall}$ in the context of singular/plural NP complements. But $Q_{\forall}$ can also combine with plural DPs in many languages, yielding a [−dist] interpretation just as with plural NPs. This is the case, for example, in Dagara (9) or in English (83):

(83) All the students read three books (between them).

The pre-theoretical intuition developed in this paper correctly leads us to expect a [−dist] semantics here, since definite plural DPs are taken to pick out the unique maximal plurality in the NP-extension.

(84)
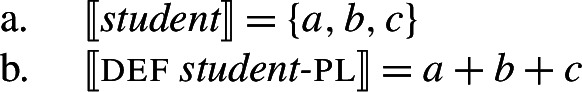

However, when directly applying our analysis to definites, two complications arise—one is purely technical, the other is an empirical issue.

The technical complication is that (84b) is of the wrong type to combine with $\mathcal{Q}_{\forall }$, which requires a type 〈*e*,*t*〉 argument. This could be fixed by assuming that plural definites are in fact of type 〈*e*,*t*〉, e.g., by letting a plural definite denote a singleton set containing the maximal plurality, (85):

(85)
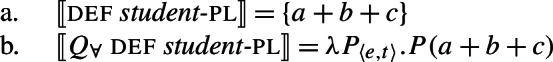

However, this raises the question of how the definite composes with the predicate in sentences without quantifiers like (86). If the definite and the predicate are both of type 〈*e*,*t*〉, this will not work.

(86) The children are awake.

To resolve this issue, we take inspiration from recent work arguing that plural predicates themselves perform existential quantification over a set of pluralities contributed by the argument DP (see Chatain 2021 and Križ and Spector 2021). As Chatain (2021) shows, this idea is motivated independently by the exceptional narrow scope behavior of definite plurals and bare plural indefinites.

There are different implementations of this idea; the exact choice is irrelevant for our purposes. For simplicity, we assume that the argument contributes the pluralities to be quantified over (unlike much of the literature, which takes them to be contributed by an operator attaching to the predicate; see Chatain 2021 and Križ and Spector 2021 for discussion). So in (86), *awake* quantifies existentially over a set of pluralities contributed by *the children*. Accordingly, definite DPs denote objects of type 〈*e*,*t*〉 and (plural) predicates are raised to type 〈〈*e*,*t*〉,*t*〉.^35^ Each plural predicate performs existential quantification over the set of pluralities contributed by its argument, as in (87):

(87) $[\!\![{}\textit{awake}]\!\!]{}_{\langle \langle e,t \rangle , t \rangle} = \lambda Q_{\langle e,t \rangle}. \exists x_{e} \in Q. ^{*}\textbf{awake}(x)$

This permits definites to compose with the predicate in the absence of a quantifier, (88).^36^

(88)

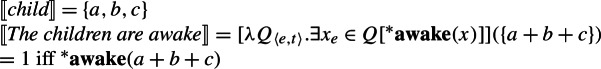

As verbal plural predicates like *awake* are now of type 〈〈*e*,*t*〉,*t*〉, we must adjust the type of the second argument of $\mathcal{Q}_{\forall }$. For each *x* among the non-overlapping maximal individuals picked out by $\mathcal{Q}_{\forall }$, $\mathcal{Q}_{\forall }$ requires the nuclear scope predicate to apply to the singleton set {*x*}. As a result, the predicate’s existential force is trivialized:

(89) $[\!\![{}\textit{$Q_{\forall }$}]\!\!]{}=\lambda P_{\langle{}\alpha , t\rangle{}}.\lambda Q_{\langle{}\langle{}\alpha , t\rangle{}, t\rangle{}}.\forall x_{\alpha}[[P(x) \land \neg \exists y_{\alpha}[P(y) \land \exists z_{\alpha}[z \sqsubseteq x \land z \sqsubseteq y] \land y \not \sqsubseteq x]] \rightarrow Q(\{x\})]$

This predicts the same truth conditions as before for *All the children are awake*:

(90)
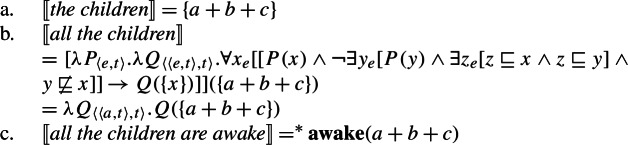

While these adjustments solve the problem of letting $Q_{\forall}$ combine with both NPs and definite DPs without a type mismatch, an empirical problem remains: The account predicts *The children are awake* and *All the children are awake* to be equivalent and therefore fails to capture the *non-maximal construals* available for definites in some languages (Brisson 1998, 2003; Malamud 2012; Križ 2015 a.m.o.).

Non-maximality is the property of plural definites by virtue of which the predicate does not always have to hold of the maximal plurality contributed by the DP. This is the case if it is irrelevant for the discourse goals whether the predicate is satisfied by the maximal plurality or a smaller one. For instance, in scenario (91), what matters for the QUD is whether *any* of the children are still awake. Here, many speakers accept an existential interpretation for (91a), while this is no longer possible if we add $Q_{\forall}$:

(91)
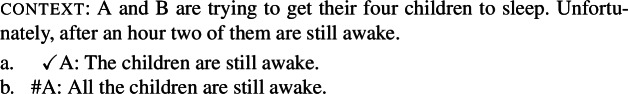

Thus, we must explain why definite plurals permit non-maximal construals and $Q_{\forall}$ blocks them. We follow Križ and Spector (2021) in assuming that non-maximal construals involve existential quantification over an upward-closed subset of the plural NP extension. “Upward-closed” means that if some plurality *x* in the NP-extension is in the subset, so are all the bigger pluralities that contain *x*. Unlike Križ and Spector (2021) and most related work, we assume that definite plurals directly denote such upward-closed sets. The exact choice of the set is determined by a parameter ⪯, a “similarity relation” between individuals that must satisfy the following constraints (cf. Burnett 2017):

(92)

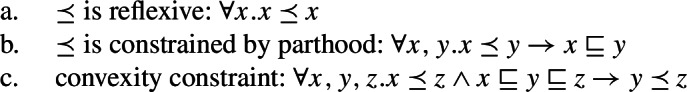

(93b) is an example of a definite DP denotation relative to this parameter. Note that if ⪯ is the identity relation, the set in (93a) will only contain the maximal individual in *P*, resulting in a maximal reading.

(93)
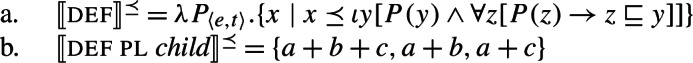

The DP denotation in (93b) produces weaker truth conditions for *The children are awake*: At least one of the pluralities in (93b) has to satisfy the predicate ^∗^**awake**.

(94)
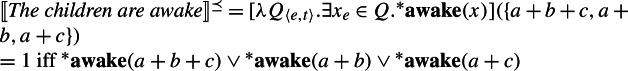

**Fig. 4:**
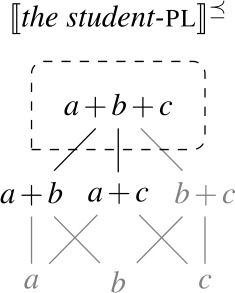
Plural DP extensions and their maximal elements

As illustrated in Figure 4, this weaker semantics for definite plurals will not affect our predictions about constructions in which $Q_{\forall}$ combines with a DP: If we assume, following Križ and Spector (2021), that a definite plural always denotes an upward-closed subset of the NP extension, this subset must contain the maximal plurality. $\mathcal{Q}_{\forall }$ then picks out this maximal plurality, giving rise to a maximal reading. Thus, even given the denotation in (93-b) for the definite, the *all*-QP will have the semantic contribution in (95):

(95)
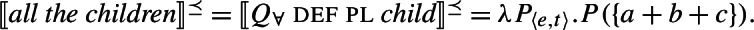

So our proposal correctly predicts that $Q_{\forall}$ blocks non-maximality.

Having raised the type of definite plurals, we also must adjust the type of the partitive operator part, which combines with a definite plural as well. The phrase headed by the definite determiner def in a structure like (96) now has type 〈*e*,*t*〉, so part must be permitted to take a type 〈*e*,*t*〉 argument.

(96) *all of the boys*
  [$Q_{\forall}$ [pl [# [part [def [pl [# [N_*PRO*_ $\sqrt{\textsc{boy}}$]]]]]]]]

We revise the lexical entry for the partitive as in (97). Its presupposition, which requires the type 〈*e*,*t*〉 argument to be a singleton set, encodes the partitive constraint. (So when 〚part〛 combines with a definite plural, the presupposition will be met only if the definite plural receives a maximal interpretation.)

(97) 〚part$]\!\!]{}=\lambda P_{\langle e,t \rangle}: |P| = 1. \lambda x. \exists y [y \in P \land x \sqsubseteq y ]$ revised

The semantics in (97) blocks partitives from combining with bare (singular or plural) NPs: While the latter will also denote sets of type 〈*e*,*t*〉, they will always have more than one element (except in cases where there is only a single individual that has the property in question).

### Number and definiteness in [−dist] UQ complements

We now have a semantic account of UQ with plural DP complements. What it leaves unanswered though is why it is so common cross-linguistically for non-distributive UQs to combine with a plural DP rather than an NP. In languages like Dagara or Arabic, a DP complement is even required for non-distributive UQ. On our semantics, 〚*boys*〛^⪯^ and 〚def *boys*〛^⪯^ have the same maximal element whenever both are defined. Therefore, apart from the existential presupposition it triggers, the determiner seems to be semantically redundant below $Q_{\forall}$. We will address this issue in two steps.

First, let us reconsider whether the determiner is indeed semantically redundant. As discussed by Matthewson 2001, the difference between DP and NP complements of [−dist] UQs in English seems to correlate with a difference between what we’ll term ‘situationally restricted’ and ‘unrestricted’ quantification. For instance, (98), where the domain of quantification is explicitly restricted, seems to require the presence of the definite determiner. In contrast, uses of UQ to express generalizations that are not situationally restricted may lack an overt determiner, (99).

(98) #All pages in this book are torn.
  (Partee 1995, 583, cited in Matthewson 2001, 169)

(99) All swans are white.

While this distinction between “situationally restricted” and “unrestricted” quantification is hard to pin down (just like the related concept of genericity, to which the interpretation of [−dist] UQ with NP complements has been linked (see Gil 1995, Löbner 2000, fn. 38, Gajewski 2005, 113)), the contrast suggests that the presence/absence of D in [−dist] UQ contexts has a semantic effect after all.

This still leaves us with several questions: First, it fails to explain why in languages like Dagara or Arabic [−dist] UQs *require* DP complements. Given the standard semantics for plural NPs we have assumed throughout, even a situationally unrestricted domain, when closed under sum, should provide us with a maximum, so nothing should prevent [−dist] UQs from combining directly with plural NPs, yielding non-distributive quantification.^37^ Second, it doesn’t explain why languages like German permit [−dist] UQ structures without any surface realization of definite D to be situationally restricted, as shown in (100) (the same construction would be used to express generalizations like (99)).

(100)
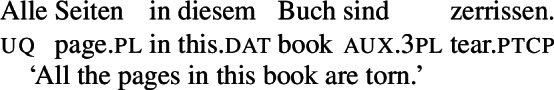

A third, related question is why “situational restrictedness” is not simply encoded by the quantificational element itself, but rather in a separate element, namely, the determiner.

While we must leave the first issue open, Haslinger (2024b,a) gives a pragmatic account to the latter two issues. Nothing in this paper hinges on the correctness of this proposal, but we give a very brief sketch to show that the variation between the UQ+NP constructions in English and German can be captured within our approach.

The basic idea in Haslinger (2024a) is that interacting pragmatic constraints force precise sentences to be structurally more complex than their imprecise alternatives. “(Im)precision,” very roughly, refers to the extent to which the meaning of the sentence can vary depending on the QUD. For example, (101a) is “precise” in the sense that its semantics does not depend on the QUD-dependent ⪯-parameter, and has the “imprecise” alternative in (101b), which does depend on ⪯.

(101)
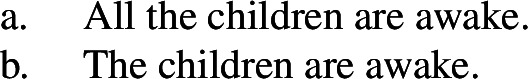

Haslinger (2024a) claims that sentence pairs like (101) are subject to pragmatic competition involving two submaxims of the Gricean maxim of Manner: A preference for structures that are less complex in the sense of Katzir (2007), which would favor (101b), and a preference for sentences that are more precise, which would favor (101a). This competition derives a systematic complexity asymmetry between precise and imprecise expressions: In a language where (102b) is an imprecise alternative of (102a), (102b) would violate Manner, because (102a) is preferable in terms of precision and not worse in terms of structural complexity.

(102)
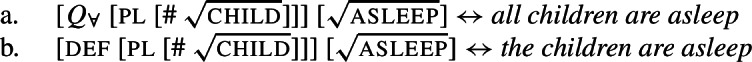

To avoid this outcome, “situational restrictedness” must be encoded in an element separate from $Q_{\forall}$, thus making the UQ sentence structurally more complex than its definite-plural alternative. One way of ensuring this is to add a D head below the UQ, resulting in structure (103).

(103)
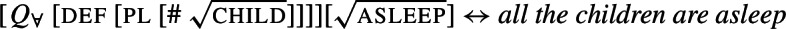

But how do we analyze languages like German that do not require overt definite determiners in situationally restricted [−dist] UQ (104a)? At first sight, the proposal would lead us to expect that (104a) will block the definite-plural alternative in (104b).

(104)
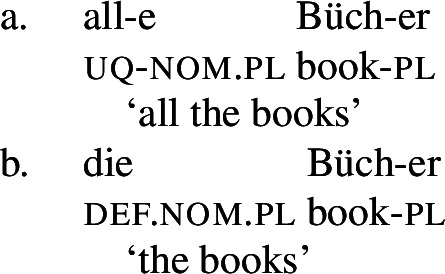

Following Haslinger (2024a), we suggest that in such languages, QPs like (104a) contain a D layer below the quantifier, so that *all-* in (104) is a portmanteau realization of Q and D. This raises the problem that it is sometimes possible for German *all-* to occur in the absence of D, e.g., in *alle von den zehn Büchern* ‘all of the ten books.’ We therefore want *all* to spell out the span $[Q_{\forall} [D]]$ if both heads are present, but to also be able to realize $Q_{\forall}$ in the absence of D. This can be done using the formal device of a pointer, introduced in Sect. 6.2. The lexical entries for the German UQ forms are then as follows:^38^

(105)
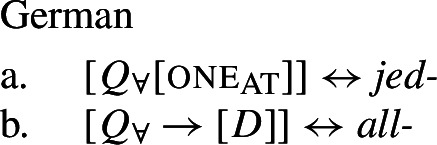

In other words, situationally restricted UQs with plural complements must be structurally more complex than their imprecise alternatives so as to satisfy a pragmatic requirement. In languages where these imprecise alternatives are definite DPs, non-distributive UQ phrases must thus be structurally more complex than a DP, a requirement that can be satisfied by inserting a determiner below the UQ. We speculate that the definite determiner is the only element licensed in this position that guarantees a non-distributive interpretation of the UQ.

In sum, we propose, following Matthewson (2001), that in languages that overtly realize definite D, the complements of situationally restricted [−dist] UQs are consistently DPs. (Since the proposal summarized above relies on the relative complexity of UQs and imprecise non-quantificational plurals, it is agnostic with respect to the universality of the category D in languages that permit bare plurals in definite contexts.) One empirical advantage of this view is that it gives us a handle on the word order variation found in languages like Wolof, where the quantifier *-epp* precedes its restrictor predicate on a [+dist] use, but follows it on a [−dist] use. We will see in Sect. 7.2 that this difference cannot be directly driven by number. But if [−dist] UQs consistently take complements that are structurally bigger than an NP, it can be implemented by assuming that these complements have to move to the specifier of Q, while bare NPs do not.

## Dissociating singular number from semantic atomicity

In this section, we provide independent evidence for the view that the distribution of [−dist] and [+dist] UQ forms is driven by semantic properties rather than morphosyntactic features. We first expand on two observations already mentioned in Sect. 5.1 that were unexpected from the perspective of morphosyntactic number—the co-occurrence of [−dist] forms with mass NP/DP complements, and the ‘whole’ meaning available for [−dist] UQ forms with singular complements in some languages. We then compare our proposal to two alternative approaches to the data motivating the DNG, based on morphosyntactic number (Winter 2001) or definiteness (Fassi Fehri 2020).

### UQ with mass complements

Several languages in our sample have [−dist] UQ forms that can be used with mass complements. While [+dist] UQs combining with mass nouns are also attested, these cases seem to systematically involve coercion of the mass noun into a count predicate with atoms corresponding to portions or subkinds. This pattern is exemplified for English, Basque, and Imbabura Quichua in (106)-(108) (see Table 1 above for an overview of [−dist] and [+dist] UQs in these languages).^39^

(106)

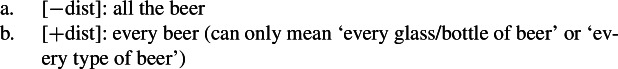

(107)

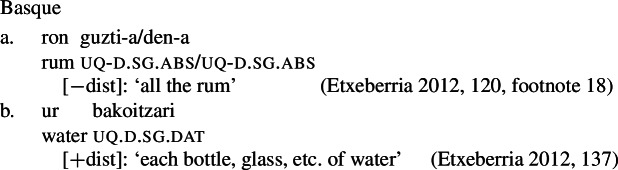

(108)
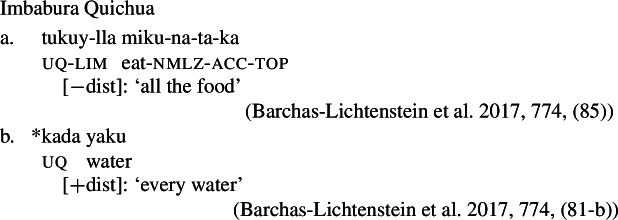

Since the mass complements in the (a) examples trigger singular agreement, the [−dist] UQ forms are puzzling for the view that the choice between UQ forms is conditioned by morphosyntactic number. In contrast, from our semantic perspective, these facts are unsurprising: The one-elements in the structure of [+dist] forms require a predicate true of atomic or non-overlapping individuals, and mass nouns are typically modeled as predicates true of overlapping “portions of matter” that cannot be divided into atomic parts (see, e.g., Link 1983). Thus, once we extend the ontology from Sect. 3.1 above to permit mass predicates, these predicates are predicted to come with [−dist] forms.

Recall that we have so far been assuming a one-to-one correspondence between individuals in $D_{e}$ and nonempty sets of atomic individuals from the set *AT*. This view does not allow us to model the semantics of mass nouns like *water*: It entails that a portion of water can always be divided into atomic subparts that can be counted, and thus predicts nouns like *water* to felicitously combine with numerals. We therefore weaken our assumption, giving up the idea that all sum individuals correspond to sets of atoms. Accordingly, the ordering ⊑ can no longer be defined in terms of set union, but must be a primitive (cf. Link 1983 who, however, distinguishes “individual parthood” for plural predication from “material parthood” for mass predication). We take $(D_{e}, \sqsubseteq )$ to be a complete semilattice with the bottom element removed, with the join operation +, which means that:

(109)
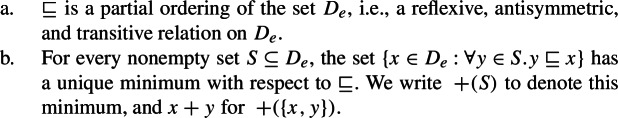

We continue to make use of a subset of individuals that are treated as “atomic” for the purposes of counting, but no longer take this subset to be structurally defined as the minimal elements of the ordering ⊑. Instead we make the following assumptions:

(110)
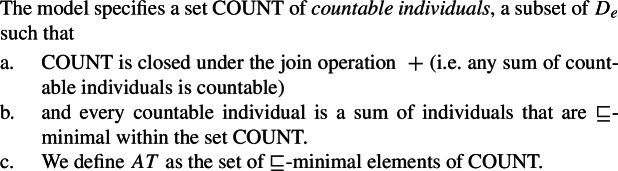

The idea is that the extensions of count predicates are subsets of COUNT, but those of mass predicates are not. Further, the individuals in the extension of a singular count predicate *P* can have parts, as long as these parts are not themselves in the extension of a count predicate.^40^ We assume following Link (1983) a.o. that the extensions of mass nouns are closed under the sum operation +, like those of plural count nouns (but unlike those of singular count nouns). So just like a sum of several pluralities that count as *children* also counts as *children*, a sum of several portions of *water* also counts as *water*:

(111) $[\!\![{}\textit{water}]\!\!]{}=\lambda x_{e}.^{*}\textbf{water}(x)$
  where **water**(*x*) is true whenever *x* is a portion of water

Given this assumption, which is standard in the literature, consider the effect of $\mathcal{Q}_{\forall }$ on mass NP and DP complements. If 〚*water*〛 is closed under sum, it has a unique maximum—the sum of all portions of water. Since $\mathcal{Q}_{\forall }$ only quantifies over portions *x* of water such that any overlapping portion *y* of water is part of *x*, it picks out this unique maximum. Thus, combining $Q_{\forall}$ with a noun like *water* results in a quantifier that applies its scope predicate to the sum of all portions of water, which seems to adequately capture the meaning of *all (the) water*.

(112)
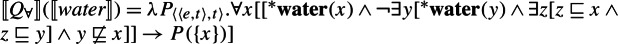

Uses of $Q_{\forall}$ with a mass DP complement are also straightforwardly captured: Applying def to the mass noun picks out a set of portions of water that stand in the contextually provided ⪯-relation to the sum of all portions of water (113). Being upward-closed, this set must have the same maximum as 〚*water*〛, so $Q_{\forall}$ has exactly the same effect it would have without the determiner.

(113) 〚def〛^⪯^(〚*water*〛)={*x* | *x*⪯*ιy*[^∗^**water**(*y*)∧∀*z*[^∗^**water**(*z*)→*z*⊑*y*]]}

Finally, note that any attempt to compose one of the items one_∅_ and one_AT_ involved in [+dist] UQ forms with a mass predicate will result in a presupposition failure, unless the predicate has been shifted to a singular count interpretation (e.g., *beer* meaning ‘portion of beer’ or ‘kind of beer’). The unacceptability of [+dist] forms with mass complements is therefore accounted for as well.

### ‘Whole’ readings of UQ with singular complements

Another type of structures in which [−dist] UQ forms consistently take singular complements are those in which the UQ phrase picks out a single atomic individual and the quantifier contributes a maximality effect similar to that of ‘whole’ in English. This is exemplified for Wolof in (114) (when used prenominally, the same UQ form yields a distributive universal interpretation). Similarly, in Hindi, the [−dist] UQ form *saar-* seems to contribute the meaning of ‘whole’ when combining with a singular complement and takes singular agreement (115).

(114)

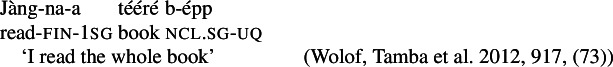

(115)
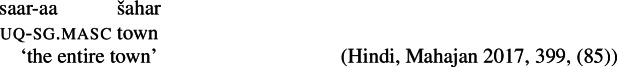

On our general approach, the difference between these structures and standard [+dist] UQs with a singular complement must be syntactic rather than lexical. Two observations provide a clue to the relevant syntactic distinction: First, in several languages, the UQs permitting a ‘whole’ reading obligatorily select for a DP rather than NP complement, as illustrated for Modern Greek in (116). Second, in our sample, the interpretation of $Q_{\forall}$ elements combining with a singular DP complement is generally non-distributive.^41^

(116)
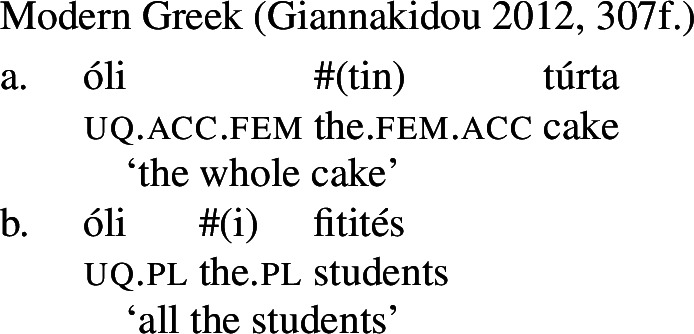

We thus suggest that these ‘whole’ structures result from combining $Q_{\forall}$ with a singular DP rather than a singular NP; (117) gives the relevant functional sequence. (We ignore the possibility of D and Q agreeing in number with the noun, which would give rise to additional *ϕ*-feature layers.) As the Hindi and Wolof examples above show, the D element in such structures does not have to be realized overtly.^42^

(117)
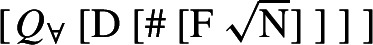

One benefit of this hypothesis is that it explains the semantics of the construction, particularly the lack of distributivity. We will first show how structures like (118) with a singular DP and no one-element are interpreted, and then discuss why one cannot be inserted.

(118)
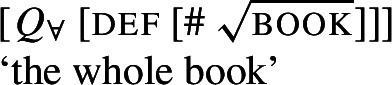

The #P in (118) denotes a predicate that is true only of atomic individuals (119). This predicate must compose with the definite determiner meaning, repeated in (120).

(119) $[\!\![{}\#P]\!\!]{}=\lambda x_{e}.x \in AT \land \textbf{book}(x)$

(120) 〚def$]\!\!]{}^{\preceq } = \lambda P_{\langle{}e, t\rangle{}}.\{x\ |\ x \preceq \iota y[P(y) \land \forall z[P(z) \rightarrow z \sqsubseteq y]]\}$

Due to the use of the *ι*-operator in (120), the definite determiner introduces the presupposition that the extension of the predicate it combines with has a unique maximal element. The following variant of the lexical entry makes this presupposition explicit:

(121) 〚def$]\!\!]{}^{\preceq } = \lambda P_{\langle{}e, t\rangle{}} : \exists !y [P(y) \land \forall z[P(z) \rightarrow z \sqsubseteq y]] . \{x\ |\ x \preceq \iota y[P(y) \land \forall z[P(z) \rightarrow z \sqsubseteq y]]\}$

Combining this with the singular predicate extension in (119), we obtain the DP-denotation in (122):

(122) 〚def$\ \#\ \sqrt{\textsc{book}}]\!\!]{}^{\preceq } = \{x\ |\ x \preceq \iota y[y \in AT \land \textbf{book}(y)]\}$
  defined only if ∃!*y*[*y*∈*AT*∧**book**(*y*)]

This DP meaning introduces the presupposition that there is a unique atomic book (for the connection between this presupposition and maximality in the semantics of plural DPs, see Sharvy 1980). But unlike the standard analysis on which singular definite DPs are referential, this account assigns the DP a set of type 〈*e*,*t*〉. This set depends on the contextually provided similarity relation ⪯ and consists of those parts of the unique book that count as ‘similar enough’ to the whole book for the contextually relevant purposes.^43^ This is unproblematic, as sentences with singular definites permit non-maximality: (123a) can be true if the cover of the book is mostly blue but has negligible white parts; (123b) can be true if the book contains some lengthy quotes in English.^44^

These effects fall out from the truth conditions our approach generates, which are schematized in (123c).

(123)
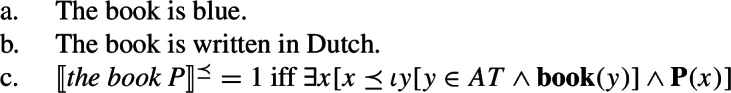

Being of type 〈*e*,*t*〉, the denotation of a singular DP can now combine with $\mathcal{Q}_{\forall }$. The QP as a whole ends up applying the predicate to the maximal element of the DP denotation, which is the entire book.

(124) $[\!\![{}Q_{\forall }\ $def$\ \#\ \sqrt{\textsc{book}}]\!\!]{}^{\preceq }=\lambda P_{\langle{}\langle{}e, t\rangle{}, t\rangle{}}.P(\{\iota x.x \in AT \land \textbf{book}(x)\})$ defined only if ∃!*y*[*y*∈*AT*∧**book**(*y*)]

Finally, let us consider what goes wrong if one is inserted on top of a singular DP. Our semantics for one comes with the presuppositions that the predicate it combines with is true of more than one individual, and that the individuals it is true of do not overlap. The denotation in (122) can never satisfy both of these presuppositions: If ⪯ is the identity relation, there is only one individual in the DP extension, but if ⪯ is more permissive, the individuals in the extension will necessarily overlap.

In sum, the denotation we posited for $Q_{\forall}$ to capture its compatibility with plural NP and plural DP complements extends to mass complements and singular DP complements and derives the correct semantic effects. The crucial semantic property that sets these three complement types apart from singular count NPs is that their extensions are closed under sum and thus contain a unique maximum. The contrast between singular count NPs, which take [+dist] UQ forms in 2-form languages, and singular count DPs, which do not, poses a problem for any direct interpretation of the DNG in terms of morphosyntactic number.

### Semantic properties vs. syntactic number and definiteness

As the above discussion illustrates, our approach to the DNG and the distribution of [+dist] forms does not directly rely on number: First, a distributive interpretation arises only if the restrictor predicate has more than one maximal element. Second, to license a [+dist] form, the extension of the restrictor predicate must not contain any overlapping individuals. Both properties distinguish singular count NPs from plural NP/DP complements, but there are systematic mismatches between these properties and morphosyntactic number: Mass NP/DP complements pattern with plurals despite mostly taking singular agreement. Moreover, the DP complements in examples like (116-a) are singular, but license neither distributive interpretations nor [+dist] forms. Our proposal therefore crucially builds on algebraic properties of the complement’s denotation, rather than its syntactic features.

In this section, we compare this semantic approach to the DNG with two alternative views from the literature, which assign a crucial role to number and definiteness respectively, and account for much of the data discussed in this paper. We will not be able to conclusively refute these alternatives, but we believe that our approach provides a more straightforward account of the overall data pattern.

#### Winter (2001)

Winter (2001) proposes an account of the DNG and the distribution of [+dist] forms that is also partly semantically based, but assigns a more crucial role to syntactic number. In his system, there are no sum individuals in $D_{e}$; pluralities are modeled as sets of atomic individuals, i.e., elements in $D_{\langle{}e, t\rangle{}}$. This permits him to model the semantics of number in terms of a type distinction: Singular nouns are 〈*e*,*t*〉 predicates and can thus be true only of atomic individuals, while plural nouns are of type 〈〈*e*,*t*〉,*t*〉 (125). Collective predicates of the *meet/gather* type directly apply to sets of type 〈〈*e*,*t*〉,*t*〉.^45^

(125) $[\!\![{}\textit{student}]\!\!]{}= \lambda x_{e}.\textbf{student}(x)$; $[\!\![{}\textit{students} ]\!\!]{}=\lambda X_{\langle{}e, t\rangle{}}.X \subseteq \{x \in D_{e} : \textbf{student}(x)\}$

This type distinction allows Winter to derive the DNG as follows. The lexical meaning of $Q_{\forall}$ is uniformly classical distributive universal quantification, as in (126), with the argument type 〈*e*,*t*〉. Attempting to compose such a determiner meaning with a plural predicate (type 〈〈*e*,*t*〉,*t*〉) yields a type mismatch that must be fixed by raising the type of the determiner: The type-shifting operation *dfit*, (127)[a], takes a quantificational determiner $\mathcal{Q}$ that expresses a relation between type 〈*e*,*t*〉 predicates and turns it into a relation between type 〈〈*e*,*t*〉,*t*〉 predicates. So for a UQ phrase to combine with a non-distributive predicate of the *gather/meet* type, the quantificational determiner has to undergo *dfit* before combining with its restrictor. The semantic effect of *dfit* on the quantifier meaning in (126) is illustrated in (127)[b].

(126) $[\!\![{}\textit{$Q_{\forall }$}]\!\!]{} = \lambda P_{\langle{}e, t\rangle{}}.\lambda Q_{\langle{}e, t\rangle{}}\forall x_{e}[P(x) \rightarrow Q(x)]$

(127)
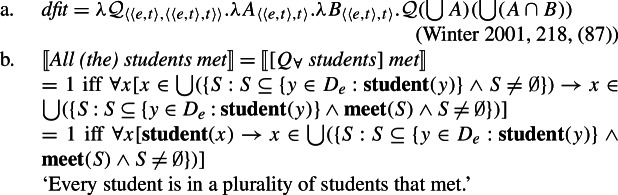

Importantly, Winter assumes that *dfit* is not freely available, but applies only if a local type mismatch must be repaired. Accordingly, it can apply only if the restrictor argument of $Q_{\forall}$ is plural, thus deriving a direct connection between non-distributive predication and plural number. On this view, [+dist] forms in 2-form languages are lexical entries for $Q_{\forall}$ that syntactically require a singular complement.

While Winter’s approach derives the DNG, it faces problems with mass complements and singular count DP complements: The assumption that plural predicates are of a higher type than singular predicates is crucial in deriving non-distributive readings of quantifiers, so to capture non-distributive UQ with mass complements, mass nouns must be treated as predicates of pluralities, i.e., 〈〈*e*,*t*〉,*t*〉 predicates. Mass entities thus must be conceptualized as having atomic parts; for example, the extension of *water* is a set of sets of water ‘atoms’ (see e.g., Chierchia 1998). While counterintuitive, this derives the non-distributive *interpretations* of UQs with mass arguments. But then, why do we not find [+dist] *forms* with mass complements if their distribution is conditioned by singular number? One could assume that [+dist] forms syntactically select for a singular *count* complement, but this would raise the puzzle why, in several languages, mass nouns pattern with plural nouns for the purposes of “agreement” with the UQ, while patterning with singular nouns for the purposes of other agreement phenomena. On our approach, the form of UQs is not the result of number agreement with the complement, so this issue does not arise.

Another problem are the ‘whole’ uses of UQ forms with singular DP complements. If [+dist] UQ forms are conditioned by singular number, we should not find [−dist] UQ forms in this configuration, a prediction contradicted by examples like (115) or (116-a). The issue could be resolved by requiring the [+dist] forms to syntactically select for a singular count NP rather than just any singular predicate, but this raises the question of what to do about cases like *every ten minutes*. On the semantic side, it is unclear how non-maximal predication with singular DPs can be modeled in this system without analyzing such DPs as denoting sets of atoms as well, which would break the strict correspondence assumed between singular (semantic) number and atomicity.

#### Fassi Fehri (2020)

In addition to his distinction between two subtypes of UQs, discussed in Sect. 6.2 above, Fassi Fehri’s 2020 syntactic work on UQ makes a second major claim: The variation between distributive and non-distributive interpretations is driven by definiteness rather than number.

Fassi Fehri (2020) motivates this claim via two core observations. First, as we saw in Sect. 6.2, the Arabic UQ form *kull* has an obligatorily distributive interpretation when combining with degree-interval predicates, as in (128), although these predicates are morphosyntactically plural or dual. Second, *kull* can express obligatorily distributive quantification over groups when combining with a group noun taking plural agreement. Both data points cannot be explained by an approach based on morphosyntactic number, but can be captured under an account where [−dist] UQs require definite complements. Moreover, such an approach captures the data with singular complements from Sect. 7.2.

(128)
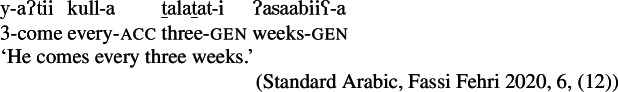

Note, however, that our semantic proposal explains Fassi Fehri’s observations without explicitly incorporating definiteness. First, group nouns have been independently argued to range over ‘group atoms’ (cf. Landman 1989), which would make them compatible with our lexical entry for one, and hence license both a distributive interpretation and a [+dist] form. Second, as discussed in Sect. 6.2, our semantics predicts distributive UQ with complements like *three weeks* to be possible, as long as the latter can be interpreted as true of non-overlapping intervals of three weeks.

The predictions of Fassi Fehri’s syntactic generalization are hard to tease apart from our semantic one, but there are empirical advantages to a view on which definite complements are not required for [−dist] UQs. First, in Sect. 6.4, we observed, following Matthewson (2001), that in English and some other languages, [−dist] UQs ranging over a situationally restricted domain must co-occur with overt determiners, while situationally unrestricted instances of UQ do not. We hypothesized that the English surface pattern reflects a structural difference between “situationally restricted” and “unrestricted” UQs, and that languages vary in whether this difference is morphologically visible. Requiring a definite complement for all instances of [−dist] UQ would make it hard to capture this systematic form-meaning correspondence. Further, our proposal allows us to remain neutral on the question of whether 2-form languages that do not require definiteness to be marked overtly at all, like Russian, must be analyzed as having null definite determiners.

Second, if definiteness is decisive for the distribution of [−dist] forms, the complement of the quantifier in phrases like *all ten of the books* must be analyzed as bearing a definite determiner. This is problematic as overt determiners in this position are unacceptable (*all the ten of the books), and neither numerals nor UQs in English can have a joint spell-out with a definite determiner in other configurations. An approach based on semantic number thus makes it easier to capture the various types of complements compatible with [−dist] UQs.

## Potential counterexamples to the DNG

Our starting point in revising the standard approach to the syntax and semantics of UQs was the empirical claim that singular count NP complements consistently give rise to distributive construals of UQs, while plural complements give rise to non-distributive construals:

(129)
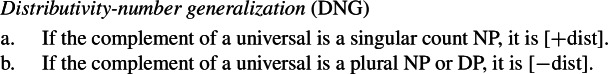

We conclude our discussion by acknowledging some problems for this number/interpretation correlation and pointing out potential ways of making some of these data compatible with our account.

The counterexamples to the DNG that we came across all concern (129b) rather than (129a), i.e., they involve [+dist] UQ with a plural complement. Within our sample, such forms seem to be present in St’át’imcets (Matthewson 1999), Gitksan (Bicevskis et al. 2017), Q’anjob’al (O’Flynn 2017), as well as in Hungarian, in the context of definite DPs with numerals (Csirmaz and Szabolcsi 2012).^46^

In St’át’imcets for example, the UQ *zí7zeg’* appears with plural DP complements, but is purely distributive relative to its nuclear scope (see Matthewson 1999; Davis 2010 for the interaction of this element with scope): it is limited to a distributive interpretation in examples like (130) and is incompatible with collective interpretations, (131).

(130)

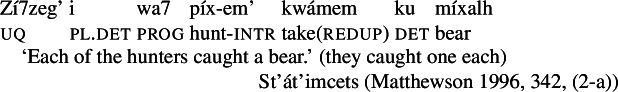

(131)
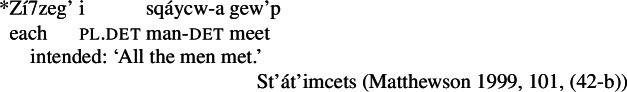

Another puzzling configuration arises in Hungarian. Most uses of UQs in Hungarian are outside the scope of our generalization, because NP complements of quantifiers are consistently number neutral (Csirmaz and Szabolcsi 2012, 402). Yet, the Hungarian UQ *mind* can sometimes combine with a definite DP containing a numeral or *az összes* (‘the all’), (132). Such DP complements must be semantically plural independently of our assumptions about number-neutral NPs. Unexpectedly, according to Csirmaz and Szabolcsi (2012), such expressions have a purely distributive interpretation, as illustrated in (132-b).

(132)
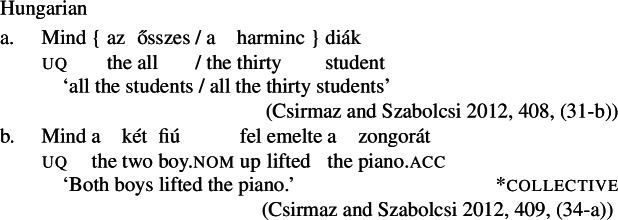

These data seem to be clear counterexamples to the DNG. However, there are two potential explanations which would render at least some of the exceptional surface patterns compatible with our generalization.

The first potential explanation is that some of these languages might lack overt partitives, in which case the exceptional constructions could involve zero partitive structures, escaping our generalization altogether. As O’Flynn (2017) claims, Q’anjob’al generally seems to lack a morphological distinction between partitives and non-partitives. Thus, we could view the exceptional UQ structures as involving a null part element. For Gitksan, we are not aware of any general discussion about partitives. However, Bicevskis et al. (2017) provide examples involving plural expressions that are translated as partitives, but do not seem to involve overt partitive marking. This could point to the option of zero partitives in Gitksan.

As for St’át’imcets, we do not have an explanation for the exceptional behavior at the moment that is in line with our account. Matthewson (1996) points out that universal quantifiers in St’át’imcets share properties with partitives, which might seem to suggest that this language, too, lacks overt partitive marking. However, this does not seem to be the case: Matthewson (2001, p. 101) provides examples where quantifiers combine with DPs including a preposition meaning ‘from’ before the determiner, suggesting that St’át’imcets does in fact have overt partitives. Thus, we leave this as a genuine counterexample to the DNG requiring further investigation.

In Hungarian as well, the presence of overt partitives renders an account of the counterexamples in terms of a zero partitive unlikely, but another potential account compatible with the DNG is available: We tentatively suggest that the unexpected number/interpretation combination in (132-b) is due to distributivity in this construction being contributed independently of the quantifier. In support of this option, we first observe that *mind* is not a typical [+dist] quantifier: Csirmaz and Szabolcsi (2012, 409f.) show that it is compatible with mass complements, and Winter (2001) provides an example with ‘gather’ (133). In our terms, this suggests that the representation of *mind* does not involve one.

(133)
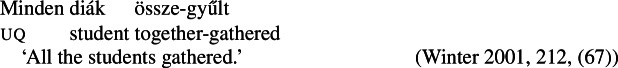

The distributivity requirement with predicates like ‘lift the piano’ might then be due to the syntactic position of the quantifier rather than its form. This is supported by existing work on the syntax of Hungarian quantifiers. As discussed by Csirmaz and Szabolcsi (2012) (‘C&S’), the literature distinguishes three regions of the left periphery where preverbal quantifiers can appear, and claims i) that *mind*-phrases are in general restricted to region 2 (C&S, p. 452) and ii) that quantifiers in region 2—independently of whether they are universal or existential—are obligatorily interpretated distributively (C&S, p. 402).

In contrast, “counting expressions” are typically associated with region 3, according to C&S, and *az összes*-DPs typically appear in region 1. If *mind* forces these DPs to appear in region 2, the distributive interpretation might be due to a distributivity operator syntactically separate from the quantifier in region 2.^47^

In line with this hypothesis, (134) shows that the *mind*+DP construction can receive a non-distributive interpretation if it occurs postverbally, even if the non-distributive predicate is not of the *gather*-type: (134) can convey that the number of poems read by the thirty students adds up to thirty.

(134)
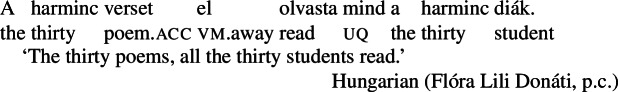

In summary, there are potential independent explanations for three of the four languages within our sample exhibiting [+dist] UQ strategies that (superficially) contradict the DNG. In Hungarian, closer inspection suggests that the quantifier form itself is not [+dist], and the distributive effect might reflect an independent requirement tied to a particular syntactic position. For Gitksan and Q’anjob’al, there is reason to suspect that these languages lack overt partitives, suggesting that the exceptional structures might involve zero partitives. St’át’imcets remains a counterexample for now. We take this to suggest that our cross-linguistic semantic proposal based on the DNG is mostly on the right track, but that more research is needed to understand the conditions under which counterexamples with plural complements are found.

## Conclusion and open issues

Based on existing as well as new data, we gave a semantic account of a correlation previously noted in the literature between the interpretation of universal quantifiers (UQs) and the morphological number of their complement: distributive UQs tend to have singular complements and non-distributive UQs—or rather, UQs that permit non-distributive interpretations—have plural complements.

Supported by the observation that languages like Dagara use the same form for both types of quantification, with the interpretation determined by the number of the complement, we proposed a single primitive $Q_{\forall}$ underlying both distributive and non-distributive universal quantification cross-linguistically, contrary to standard assumptions. Appealing to the different semantic properties of singular and plural nouns, we argued that the distributive and non-distributive meanings follow from the composition of $Q_{\forall}$ with a singular or plural noun, respectively: $\mathcal{Q}_{\forall }$ requires the scope property to hold of every maximal element of its complement’s denotation. If the latter contains pluralities, the effect will be non-distributive quantification, but if it contains only atoms, this will result in distributive quantification. We argued that this approach also works for complements containing numeral-modified indefinites if the maximal elements are required to be non-overlapping, and derives the correct effects for UQ with definite complements.

Since strategies involving a single UQ form for both types of quantification are the default case under our approach, the formally different distributive and non-distributive UQs in languages like English required further explanation. Based on i) cross-linguistic structural complexity asymmetries between forms associated with distributive and non-distributive quantification and ii) the frequent occurrence of morphemes formally identical to the numeral ‘one’ in [+dist] forms, we suggested that distributive quantification involves extra structure: we posited additional heads one_∅_ and one_at_ that apply right below $Q_{\forall}$ in such languages and presuppose non-overlap and atomicity respectively. In some languages, these items can be spelled out together with $Q_{\forall}$, resulting in two different forms for [+dist] and [−dist] UQs. Further, a language can realize these two items differently, resulting in formal and semantic variation between [+dist] UQ forms.

The last step was to point to a number of potential counterexamples to our distributivity-number correlation, where purely distributive UQs take plural complements; we suggested ways of making some (although not all) of them compatible with our account.

We end by discussing a few open problems in the morphosemantics of universal quantifiers that seem particularly interesting for future work. A much broader question for future research is, of course, whether the correlation between semantic number and distributivity extends to other classes of quantifiers^48^—and, if so, whether our account can be extended to such cases.

### Floated quantifiers

While we focused on quantifiers internal to nominal arguments, any structural proposal about distributive and non-distributive quantifiers should ultimately be extended to floated UQs (Sportiche 1988; Bobaljik 2003 a.o.). One problem for our proposal is that in some 2-form languages, the morphological number of the antecedent of floated [+dist] UQs is plural, as illustrated for German in (135).

(135)
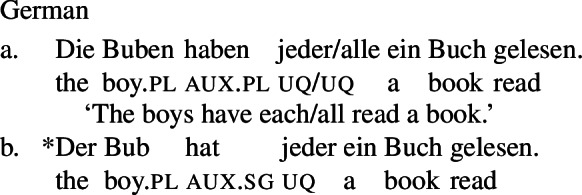

One might hypothesize that the spell-out and the interpretation in these constructions depend on a (singular or plural) pro-NP in a silent partitive structure in the floated position. This is supported by the observation that English *every*, which unlike *each* cannot combine with partitives, also cannot be floated. But this hypothesis faces an obstacle: In German, floating with a DP antecedent is possible only for UQs, as shown by the contrast between (135a) and (136a), but if the antecedent is a full partitive structure, non-universals can also be floated, (136b). If (135-a) involved a covert partitive structure, the set of quantifiers that can be floated should be the same as with overt partitive antecedents. Accordingly, the exceptional behavior of $Q_{\forall}$ in floated constructions remains an open issue for now.

(136)

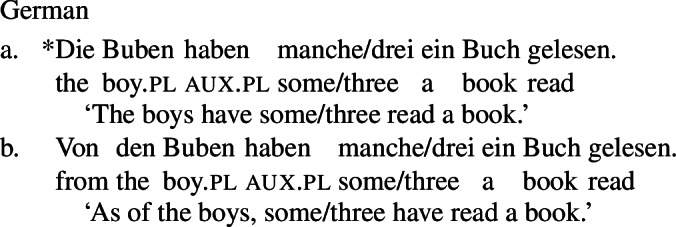

### Exceptional uses of [+dist] forms with definite determiners

Our analysis predicts that UQ forms with a singular count DP complement get a ‘whole’ reading rather than a distributive interpretation, and that we systematically find the [−dist] forms of quantifiers in this construction (Sect. 7.2). However, some languages do have structures in which [+dist] UQ forms co-occur with a definite article. An example of this is the Greek *o káthe* construction (137), discussed in detail by Giannakidou (2004) and Lazaridou-Chatzigoga (2015). A related structure is found in Basque (Etxeberria 2005, (37a,b)).

(137)
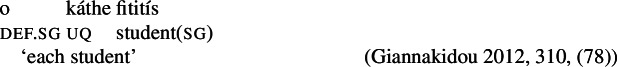

Our proposal does not account for these structures. At first sight, they might seem to support a decomposition of [+dist] UQs that contains a definite determiner element in the scope of the UQ. This is not a new idea; for instance, Sauerland 2003 and Kallulli and Rothmayr 2008 propose decompositions of German *jed-* into an independently attested distributive element *je* and the definite determiner *d-* (although see Zimmermann (2002, 51) for the point that this decomposition does not match the diachronic origin of *jeder*). However, assuming the underlying merge order [Q [D NP]] for [+dist] UQs would make these structures parallel to [−dist] UQ structures, which we think would be a mistake for two reasons. First, in Greek, the linear order of the exceptional [+dist] constructions is D-Q-NP, which does not match the word order of the Greek [−dist] UQ.^49^ Second, it would fail to explain why [+dist] forms that obligatorily co-occur with a definite determiner are typologically uncommon, while this is a very common pattern for [−dist] forms.

However, these arguments do not exclude the possibility that [+dist] UQ forms involve a D element in a different position—e.g., forming a constituent with the quantifier, as Giannakidou (2004) proposes for Greek, or merging above it, as Leu (2009) proposes for Greek *o* and the *d-* element in German *je-d-er*.

The main puzzle for accounts of this type is what the semantics of a D head merging with or above the UQ would be. Existing semantic proposals for (137) involve highly nonstandard meanings for the definite determiner; for instance, Giannakidou (2004) suggests that the definite determiner *o* composes directly with the UQ *káthe* to form a complex UQ by incorporation. As Lazaridou-Chatzigoga (2015) notes, this does not provide a satisfactory compositional account, since it is unclear how to reconcile it with other uses of the definite article. However, Lazaridou-Chatzigoga’s 2015 own account faces a similar problem on our view: She observes that the determiner adds a strict event distributivity requirement, comparable to what has been reported for *each* as opposed to *every* (Tunstall 1998). Motivated by this, *o* is interpreted as an identity function with an additional presuppositional requirement, which combines with a distributive quantifier and preserves its type. Its type and semantic function in the *o káthe* construction therefore differ from regular definites.

Similarly, Leu (2009) argues on syntactic grounds that the *d-* element in German *je-d-er* is a complementizer-like element in an extended adjectival projection of the UQ element, with a silent D head merging on top of this projection, so that the underlying merge order is [D_∅_ [*-d* [⋯ [Q [⋯ NP]]]]]. On this proposal as well, the *d-* “complementizer” must differ from a regular definite D semantically, and the silent D head must have a nonstandard meaning to allow the QP to scope out.

In sum, we currently see no way around the conclusion that (137) involves a semantic primitive different from standard accounts of the definite determiner; the puzzle then is why this element and the definite determiner can have the same spell-out. The phenomenon might suggest that the semantic contribution standardly assigned to definite determiners can be decomposed into several elements, only one of which can merge on top of Q. We leave the exploration of this idea to future work.

## Abbreviations

abs: absolutive,
acc: accusative,
aff: affirmative (polarity marker),
aux: auxiliary,
dat: dative,
def: definite,
det: determiner,
erg: ergative,
fem: feminine gender,
fin: ‘head of FinP’ (in examples taken from Tamba et al. 2012),
gen: genitive,
indef: indefinite,
intr: intransitive,
masc: masculine gender,
ncl: noun class marker,
neut: neuter gender,
nom: nominative,
pfv: perfective,
pl: plural,
prog: progressive,
prox: proximal,
redup: reduplication,
refl: reflexive marker,
sg: singular,
uq: universal quantifier,
vm: verb particle,
3: 3rd person

## Footnotes

2 The sample this generalization is based on—taken from Keenan and Paperno (2012), Paperno and Keenan (2017), our own elicited data, our native-speaker judgments and other papers on individual languages (see below)—contains 16 languages that mark number on nouns and show this pattern: Adyghe, Basque, Dan-Gwɛɛtaa, English, German, Modern Greek, Hausa, Hindi, Italian, Logoori, Persian, Imbabura Quichua, Malayalam, Russian, Telugu, Turkish. Further, it contains 6 languages with number on nouns that show the pattern discussed in Sect. 2.2, which also supports our subsequent generalization relating the interpretation of UQs to number: Standard Arabic, Syrian Arabic, Dagara, Gourmantchema, Moore and Wolof. We came across potential counterexamples (to be discussed in more detail in Sect. 8) from 4 languages: Gitksan, Hungarian, Q’anjob’al, and St’át’imcets. Throughout the paper, we will also occasionally discuss data points from the literature on other languages. We rely on native-speaker judgments for Dagara (one author is a native speaker) and German (four of the five authors are native speakers), and on judgments we elicited for Moore, Gourmantchema, and Syrian Arabic. Otherwise, we made use of existing literature—Fassi Fehri (2020) for Standard Arabic, Zimmermann (2008) for Hausa, Landman (2016) for Logoori, Matthewson (1996, 2001) for St’át’imcets and several sources, including Partee (1995), Winter (2001), Matthewson (2001) a.o. for English. For the other languages, we relied on the descriptions in Keenan and Paperno 2012; Paperno and Keenan 2017. For the purposes of testing this generalization, we excluded from consideration languages that systematically allow nouns to be number-neutral, or where the descriptions in Keenan and Paperno (2012), Paperno and Keenan (2017) are not informative enough about the semantic behavior and syntactic requirements of the relevant quantifiers.

3 For instance, a reviewer points out that in Hungarian, non-distributive UQ is possible with what looks like a singular count NP complement:

Yet, NP complements of quantificational elements in Hungarian are generally unmarked for number. Since this extends to the complements of numerals like ‘two,’ which presumably have plural individuals in their extension given the standard semantics of numerals, we assume that the absence of number marking on the quantifier complement in Hungarian does not necessarily indicate that the complement is semantically singular. As our analysis for number languages will rely on the semantics of number rather than morphosyntactic number agreement, it could in principle be extended to languages like Hungarian.

4 See Schein 1997; Kratzer 2003; Ferreira 2005; Champollion 2010; Haslinger and Schmitt 2018; Chatain 2021 a.o. on complications regarding UQs in object position.

5 We will argue below that the distributive interpretations available with *all*-type quantifiers are not tied to the quantifier, but due to distributivity operators within the predicate it combines with.

6 In German partitive structures, *jed-* can combine with a genitive plural DP complement, or a PP containing a dative plural DP. We put these constructions aside here and return to them in Sect. 5.6, where we will assume that these plural DPs are not direct arguments of the quantifier, so that there is no genuine violation of the distributivity-number generalization introduced in (17) below.

7 We use the label “1-form languages” for languages that have *some* UQ strategy of this kind, regardless of whether it is the only strategy; a 1-form language may have additional UQ forms specified as [+dist] or [−dist]. Since our account of the distinction between 1-form and 2-form strategies will involve variation in the availability and postsyntactic realization of particular functional heads, rather than language-level parameters, it is compatible with the co-existence of both strategies within a language.

10 Tamba et al. (2012) report that some, but not all speakers accept *-epp* with a DP argument in addition to the NP strategy illustrated in (14-b).

11 Francez and Goldring (2012) suggest Hebrew *kol* behaves analogously, but see Bar-Lev and Margulis (2014) for data that complicate the picture and suggest that the different uses of *kol* are better captured in terms of distinct lexical items.

12 Competing proposals assume that the denotations of plural nouns contain no atomic individuals (see, e.g., Link 1983; Chierchia 1998). The choice between the two types of theories is irrelevant for our point here.

13 Link’s 1983 more involved analysis of *all*, which makes use of a “partake” operator to capture its maximality inferences, would also be subject to the criticism discussed below in the main text.

14 To our knowledge, this meaning is unattested in natural languages. It is unclear why, since it would in principle give rise to non-trivial truth conditions. See Link (1987) for related discussion.

15 According to Khanjian (2012), Western Armenian also permits *amen* without the numeral *meg* as a [+dist] UQ. From their description, it is not clear whether adding *meg* makes a semantic difference.

16 We would like to thank one of the anonymous NLLT reviewers for very helpful comments on this section.

17 In an earlier manuscript version of this paper, we presented the spanning approach as a variant/subtype of Nanosyntax. While this framing is adopted in some recent work (e.g., Taraldsen 2018), most of the literature reserves the term Nanosyntax for approaches in which the lexicon stores trees, not just spans. We thank a reviewer for pointing this out.

18 The standard intersective entry for the numeral *one* is given in (40):

One might try to unify one and the numeral *one* by claiming that *one* is semantically unlike other numerals, but the proposal in the main text, on which there is no synchronic derivational relation between the two, strikes us as less problematic.

19 We leave the exact semantic role of the conjunction and disjunction markers in (43) for further research. The conjunction marker is unsurprising given the general tendency for UQ morphemes to surface in the structures of distributive conjunctions; see Sect. 4. But if *-um* is a spell-out of $Q_{\forall}$ as this suggests, its appearance in the morphology of non-universal quantifiers needs to be explained (Jayaseelan 2011). Further, the role of the disjunction marker (*-oo* in (43)), which is cross-linguistically uncommon in UQs, is not well understood. However, Jayaseelan (2011, 283, fn. 24) mentions an alternative parse of the form *oor-oo* as a reduplicated form of *oru* “one” that fits better into our theoretical picture.

20 Our claim that [+dist] forms in 2-form languages can either be decomposed into an exponent of $Q_{\forall}$ and one of one, or spell out $Q_{\forall}$ together with one, predicts that transparent morphosyntactic containment should be quite common cross-linguistically. Yet, while Gil (1995) points out that the [+dist] forms are often morphologically based on the [−dist] forms, he also notes that there is often no productive process deriving the former from the latter (see, e.g., Georgian in Sect. 5.1). We have nothing to say about why unproductive derivations are not found more often, and why transparent containment as in, e.g., Western Armenian is not more common.

21 We remain neutral on whether the higher *ϕ*-feature layer is present at the level forming the input to semantics. However, it is crucial for us that unlike in, e.g., Sauerland’s 2003 system, the *lower* *ϕ*-feature layer must have a semantic contribution rather than being a mere reflex of agreement.

22 Leu (2009) proposes to capture this parallelism by analyzing forms like *jed-er* as extended adjectival projections. On this view, a [+dist] UQ phrase contains a silent determiner merged above the adjectival stem *jed-* and the functional projections associated with it. While this is a plausible idea, we refrain from fully implementing it here because it is unclear what the semantics of the silent D head should be, given that it would have to take a quantifier argument (see Sect. 9.2 for a related open issue).

23 We assume that languages like English and Dagara, where the UQs do not exhibit overt number agreement (see Sect. 5.3), either lack the agreement projections above $Q_{\forall}$ or realize them as null.

24 The Nanosyntax literature provides an interesting way of structurally distinguishing prefixes and suffixes, based on the assumption that the failure to spell out a given subtree can trigger movement; see, e.g., Caha et al. (2019), Taraldsen (2018) for discussion. We leave open how this idea could be combined with our analysis of UQs.

25 In particular, it is unclear to us how to implement the relevant movements in a way that is still restrictive enough to account for typological generalizations about word order within the extended NP (e.g., Cinque’s 2005 generalization of Greenberg’s Universal 20).

26 Hindi has more UQ strategies than those in (58). We do not attempt to account for all of them. It is unclear from the description in Mahajan (2017) if there are meaning differences between the various [+dist] strategies/ [−dist] strategies.

27 There are some languages in which the pro-N seems to be overt. For instance, French has the form *chac-un*, which decomposes into the [+dist] UQ form *chaque* (which is arguably itself internally complex, cf. Leu 2009) and the element *un* ‘one.’ Since the latter appears (i) in the absence of a complement with a lexical noun and (ii) in partitive constructions, but does not (synchronically) appear with lexical NP complements, it is most plausibly analyzed as a pro-N (as in English *every-one*), rather than a spell-out of the one-element internal to [+dist] UQ forms.

28 Since part denotes a function that takes only individuals as its complement, it directly encodes the partitive constraint (see Jackendoff 1977; Barwise and Cooper 1981 a.o.).

29 This is not to say that atomicity is the only semantic difference between these subclasses of UQs; see, e.g., Tunstall (1998) for differences wrt interaction with event quantification, and Matthewson (2001) for distributional differences. We leave a closer investigation of the semantics of [+atomic] UQs to future work.

30 One might ask whether this departure from the view that functional sequences are universal is necessary. The idea, briefly discussed in Sect. 5.3, that a pragmatic principle forces the insertion of one-elements whenever the presuppositions they introduce are met, would allow us to avoid this conclusion. On this view, $Q_{\forall}$ with predicates of atomic individuals would have to obligatorily co-occur with both one_at_ and one_∅_ in both English and German. We could then make use of the “pointer” idea presented below in the main text to analyze German *all-* as being able to spell out either $Q_{\forall}$ or the combination of $Q_{\forall}$ and one_∅_. In contrast, English *all* would be unable to spell out one_∅_, preventing distributivity in *all ten minutes*.

One problem for this approach is that forcing one_at_ to appear whenever it is semantically permitted to appear would lead us to expect that predicates of atomic individuals obligatorily take *each* rather than *every* in English. But since there are additional semantic properties distinguishing *every* and *each* (see, e.g., Tunstall 1998), it might be the case that the representation of *each* involves additional primitives absent in *every*, which would take care of this problem.

31 A reviewer points out that in some varieties of German, the [+dist] form *jed-* co-occurs with *ein*, which is homophonous with the numeral “one” and the singular indefinite article (Pafel 1994; Kallulli and Rothmayr 2008; Leu 2009 a.o.), (80).

This could be taken to reveal a one-element in [+dist] UQ and suggest that *jed*- is a portmanteau of $Q_{\forall}$ and a third structural primitive distinct from one_at_. We leave the analysis of this construction for future work, as there are some open problems with the view that *ein* here is an exponent of one_at_. First, given our assumption that one_at_ adds a presupposition about the restrictor predicate of the UQ, its surface position in (79), which suggests a structural position above $Q_{\forall}$, is surprising. Second, what exactly does *ein* contribute semantically in this construction? Kallulli and Rothmayr (2008) claim that it has semantic effects analogous to the use of *each* as opposed to *every* in English. If so, this might suggest that *jed*- is in fact a portmanteau of $Q_{\forall}$ and $\textsc{one}_{\emptyset}$, and *ein* the separate spell-out of one_at_. But if so, %*jede zehn Minuten* is wrongly predicted to be fine for all speakers that accept the *ein jeder* structure. This suggests there might be even more than two semantic subclasses of [+dist] UQs.

32 Corblin (2018) discusses further differences, concerning, e.g., the use of *tout* for law-like generalizations holding across possible worlds. We have nothing to say about these differences here.

33 As pointed out to us by a reviewer, this mechanism differs from the one used in Caha et al. (2019), which requires a specific lexical entry (including phonological information) rather than a subspan of features on the right-hand side of a pointer.

34 We note two further paths for future work on [+dist] UQs. First, there are other systematic differences between [+atomic] and [−atomic] UQ forms, e.g., the [−atomic] UQ forms often cannot combine with partitives (see Matthewson 2001 for English, Fassi Fehri 2020 for a cross-linguistic claim). This does not follow from our proposal and suggests more needs to be said about the structure of [+atomic] UQ forms. Second, our hypothesis that the three types of UQ form a containment hierarchy with three levels raises the question whether the UQ forms in languages with both [+atomic] and [−atomic] UQ forms follow an *ABA-pattern (Bobaljik 2012; Caha 2009), i.e., whether it is ever possible for [−dist] and [+atomic] UQ to be expressed by the same form to the exclusion of [+dist, −atomic] UQ. We currently do not have enough cross-linguistic data to test this hypothesis.

35 Any phrase traditionally assigned type 〈*e*,*t*〉 that composes with definite DPs is now assigned type 〈〈*e*,*t*〉,*t*〉, so we introduce an asymmetry between verbal and nominal predicates, with only the former being of type 〈〈*e*,*t*〉,*t*〉 and requiring existential quantification.

36 The proposal has the additional advantage of deriving an existential semantics for bare plurals without having to posit a covert existential determiner, (88) (where 〚*child*〛 = {*a*,*b*,*c*}):

37 A related question is why [−dist] UQs with NPs in English seem to prohibit cumulative construals (e.g., our initial example in (1-b) is odd if we replace *all the boys* by *all boys*). As situational unrestrictedness of *all NP*_*pl*_ leads to constraints on predicate selection (episodic predicates seem to be blocked), one might expect that changing the relevant sentences so as to express situationally unrestricted generalizations, as in (100), would license a cumulative construal relative to the predicate conjunction (see Link 1984; Krifka 1990 a.o.). Yet, judgments seem to be mixed and more investigation is needed.

38 Some speakers also accept *all die (tollen) Bücher*, literally ‘all the (great) books,’ which, as a reviewer points out, is unexpected under these assumptions. However, the meaning of *all die N* seems to differ from that of its counterpart that lacks a surface definite, *alle N*, in that the former may range over large, but non-maximal pluralities in certain contexts, while the latter cannot. Descriptively, this property of German *all die N* is shared by UQ structures containing demonstratives in English, such as *all those N*. We therefore speculate that this structure involves a use of *die* as a demonstrative rather than a regular definite determiner. If so, a proper syntactic analysis of these constructions would have to appeal to the syntax of demonstratives.

39 Tamba et al. (2012) show that in Wolof—a 1-form language according to our classification—UQs with mass complements bear a plural noun class marker, although the noun class marking on the mass noun is singular (i). This suggests that, like the choice between [−dist] and [+dist] UQs, noun-class marking on the quantifier in Wolof is sensitive to the semantics of the complement rather than its morphosyntactic number.

40 This is an oversimplified view resulting in mismatches between ⊑ and pre-theoretical notions of parthood: A person *x* and *x*’s left hand must both be minimal elements of COUNT as both are in the extension of singular count predicates, so we cannot assume that *x*’s left hand ⊏ *x*. In effect, individuals in the extension of count predicates must be distinguished from the portions of “stuff” they consist of (cf. Link 1983). See, e.g., Wągiel (2021) for potential problems with this idea.

41 A reviewer points out that expressions like *all the way* or *all the while* in English fit this pattern too. We do not know, however, why the form *all* in these constructions is not blocked by *whole*.

42 Yet, it is suggestive that the postnominal position of the quantifier in Wolof is the same as with plural DP complements.

43 Since we assumed that 〚*book*〛 consists of atomic individuals only, the non-maximal parts of the book will not themselves be elements of 〚*book*〛. As far as we can see, this is unproblematic.

44 This cannot be the full story about non-maximality in non-plural predication, since ‘subatomic’ non-maximality is available with numerals, which remove non-maximality in the plural case: (123) can be true if the covers of the books in question have some white parts. We leave a deeper investigation of this issue to future work, as it does not affect our main point.

45 This use of higher predicate types (see also Bennett 1974) differs from ours: We assumed that predicates combining with plural NP/DP arguments are raised to type 〈〈*e*,*t*〉,*t*〉, but assigned type 〈*e*,*t*〉 to both singular and plural NPs.

46 Going beyond simple universals, German *beide* ‘both’ might appear to be another counterexample of this kind. (We thank a reviewer for bringing this up.) As discussed by Leu (2009) with reference to Pafel (1994), German *beide Buben* ‘both boys’ seems purely distributive. Taking this description for granted, this would be a puzzle for our account, as the complement of *beide* is plural. Yet on closer inspection, *beide* seems to permit some forms of non-distributive predication as shown in (130a), which contrasts with examples like (130b) involving a [+dist] UQ. Cumulative readings are typically absent in the case of *beide* without modifiers like *insgesamt* ‘in total’ or strong contextual support, but this also holds for [−dist] UQ forms like English *all* (see footnote 1). The behavior of *beide*+*NP* in relation to distributivity thus at least partially resembles that of a [−dist] UQ.

47 The assumption that distributivity is encoded in a different syntactic position than the DP that seems to receive a distributive interpretation is already made by Beghelli and Stowell 1997 for English and discussed by Szabolcsi 1997 for Hungarian.

48 Relevant examples with modified-numeral indefinites are discussed by Winter (2001).

49 It is also the only word order that cannot be generated from the underlying structure [Q [D NP]] via successive phrasal movement of constituents containing the head noun. Cinque (2005) argues that this kind of movement derives the surface orders of demonstratives, numerals and adjectives relative to the noun. Extended to QPs, this account leads us to expect that a structure with Q merged above D cannot have this surface order.
